# Molecular Recognition of Dental Resin Monomers by Salivary Proteins: Integrated Docking, DFT, Molecular Dynamics, and MM/PBSA Analysis

**DOI:** 10.1002/open.70332

**Published:** 2026-11-02

**Authors:** Ravinder S. Saini, Fahad Hussain Alhamoudi, Rayan Ibrahim H. Binduhayyim, Abdulkhaliq Ali F. Alshadidi, Abdullah Hassan A. Alshehri, Abdulmajeed Okshah, Ahid Amer Alshahrani, Mohamed Saheer Kuruniyan, Vishwanath Gurumurthy, Masroor Ahmed Kanji, Artak Heboyan

**Affiliations:** ^1^ Department of Allied Dental Health Sciences COAMS King Khalid University Abha Saudi Arabia; ^2^ Department of Prosthodontics College of Dentistry King Khalid University Abha Saudi Arabia; ^3^ Department of Research Analytics Saveetha Dental College and Hospitals, Saveetha Institute of Medical and Technical Sciences, Saveetha University Chennai India; ^4^ Department of Prosthodontics Faculty of Stomatology Yerevan State Medical University after Mkhitar Heratsi Yerevan Armenia; ^5^ Department of Prosthodontics School of Dentistry Tehran University of Medical Sciences Tehran Iran

**Keywords:** dental resin monomer, DFT calculation, MM/PBSA, molecular docking, molecular dynamics, salivary protein

## Abstract

An in silico investigation was performed to study the interactions between the commonly encountered resin monomers and the major salivary proteins. Bisphenyl diglycidylmethane (Bis‐GMA), hydroxyethyl methacrylate (HEMA), and triethylene glycol dimethacrylate (TEGDMA) were assessed for molecular docking, density functional theory (DFT), molecular dynamics (MD), MM/PBSA binding free‐energy calculations, and per‐residue energy decomposition. α‐amylase, lysozyme, and albumin emerged as critical predicted targets from the docking process. DFT calculations found Bis‐GMA to possess the largest HOMO–LUMO gap (8.88 eV), suggestive of reduced electronic reactivity, and TEGDMA to have the smallest gap (5.56 eV) as well as the largest dipole moment (3.88 D), indicative of increased polarity. Mean solvent accessible surface areas (SASAs) determined through MD trajectories suggest a trend of increased conformational stability with Bis‐GMA. More favorable mean MM/PBSA predicted binding free energies were calculated with Bis‐GMA, especially with albumin (−24.53 ± 5.12 kcal/mol) and α‐amylase (−17.63 ± 7.60 kcal/mol). Residue‐decomposition heatmaps displayed mixed favorable and unfavorable contributions, which were interpreted descriptively. Recognition of monomers by salivary proteins appears to correlate with molecular dimensions, hydrophobicity, polarity, and electronic properties. These observations are preliminary calculations that require experimental evidence to reach clinical or functional conclusions.

## Graphical abstract

An integrated computational framework reveals distinct interaction patterns of dental resin monomers with key salivary proteins. Bis‐GMA shows the most favorable predicted binding profile, HEMA displays weaker and more variable interactions, and TEGDMA exhibits intermediate behavior. The findings link electronic structure, molecular flexibility, and binding energetics, providing mechanistic hypotheses for future experimental evaluation of dental resin biocompatibility.

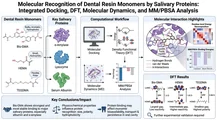

**Abbreviations**
Bis‐GMA: bisphenol a‐glycidyl methacrylate
DFT: density functional theory
FMO: frontier molecular orbital
HEMA: 2‐hydroxyethyl methacrylate
HOMO: highest occupied molecular orbital
LBD: ligand‐binding domain
LUMO: lowest unoccupied molecular orbital
MD: molecular dynamics
MM/PBSA: molecular mechanics/poisson Boltzmann surface area
PDB: protein data bank
ROS: reactive oxygen species
SDF: structure data file
TEGDMA: triethylene glycol dimethacrylate

## 1 Introduction

Resin‐based composites are commonly utilized in restorative and adhesive dentistry procedures as filling materials, sealants, and bonding agents [1, 2]. These composites consist of a highly crosslinked polymer matrix, which is synthesized from combinations of dimethacrylate monomers (generally Bis‐‍GMA or another viscous dimethacrylate monomer) and low‐viscosity comonomers (e.g., TEGDMA and HEMA) to modify handling characteristics [1, 3]. Bisphenol A‐glycidyl methacrylate (Bis‐‍GMA) is a high‐molecular‐weight dimethacrylate monomer which possesses rigid, bulky aromatic rings, which contribute to the strength of the base resin. In contrast, triethylene glycol dimethacrylate (TEGDMA) and 2‐hydroxyethyl methacrylate (HEMA) are low‐molecular‐weight dimethacrylate monomers which are more hydrophilic and less viscous than Bis‐GMA, thereby facilitating polymerization. Thus, low‐viscosity comonomers such as TEGDMA and HEMA must be used in order to dilute Bis‐GMA base resin so that they can mix and cure appropriately [3].

After a composite has cured, unreacted resin monomers leach out into saliva and other body fluids [1, 4]. Studies have shown large percentages of TEGDMA and HEMA diffuse into water or saliva following polymerization in vitro [5, 6]. One study found HEMA was released from two widely used composites into saliva, and TEGDMA was found in saliva from at least one composite formulation [7]. In simple terms, HEMA and TEGDMA are released from dental materials in higher amounts than larger monomers such as bis‐GMA. Under severe conditions, these monomers have been found in dentinal or pulp fluid at levels of about 1–8 mM, which may affect pulp cells [1, 7]. Leaching of monomer from a restoration is greatest during the first hours or days after curing and persists at reduced levels for weeks [2, 8]. Therefore, placement of a composite restoration chronically exposes saliva and soft tissues to low levels of resin monomers.

Clinical studies have also supported these findings, indicating that patients are exposed to resin monomers directly in the oral cavity following restorative procedures. Bis‐GMA, HEMA, and UDMA were identified in the saliva of patients 10 min after receiving resin‐based composite restorations. The concentration of Bis‐GMA ranged from 0.028 to 9.65 μg/mL, and HEMA ranged from 0.015 to 0.19 μg/mL. TEGDMA was also detected in the saliva of some patients. Monomer levels were no longer detected at either the 24 h or 7‐day follow‐up appointments for most patients. These data suggest that the greatest exposure to monomers occurs immediately following placement. Similar findings have been seen in more recent clinical studies, showing that the greatest release of monomers and degradation products occurs soon after placement but that some degradation products may be detected for extended time periods [9].

It should be noted that since unpolymerized monomers and oligomers can diffuse into the oral environment, they will necessarily contact salivary proteins. Some evidence suggests that resin monomers are capable of binding to or becoming sequestered by salivary proteins, thereby affecting both monomer availability and protein function. For example, measured concentrations of TEGDMA or HEMA recovered from native saliva (containing proteins) were significantly less than protein‐free controls following extraction [10]. This suggests that salivary proteins bind a significant portion of released monomers, effectively lowering the free concentration of the monomer in solution. While binding to salivary proteins may decrease monomer bioavailability (and potential toxicity), it may also disrupt protein structure/function or compete with normal protein–protein interactions. This could lead to clinical biocompatibility concerns; specifically, if resin monomers bind to proteins such as amylase or lysozyme, they could inhibit enzyme activity or provoke an immune response. Monomer–protein complexes may also increase the uptake and systemic distribution or promote immune sensitization. The extent to which these reactions impact toxicity remains unclear. Saliva contains numerous functional proteins (e.g., lysozyme, immunoglobulins, and statherin) that are involved in the maintenance of oral homeostasis [10, 11]. Modifying the function of even a fraction of these proteins with resin monomers could affect tooth remineralization, plaque formation, mucosal immunity, etc. For these reasons, interactions between HEMA, Bis‐GMA, and TEGDMA with representative salivary proteins should be investigated at the molecular level.

Direct laboratory assays of resin‐protein binding are limited. It is difficult to isolate certain protein–monomer interactions in vivo due to saliva’s complexity as well as in vitro. Molecular modeling can help fill this gap. It can be used to predict how monomers (small molecules) will dock onto proteins, estimate binding energies, and simulate dynamic processes in solution [12, 13]. Molecular docking predictions can be followed up by more explicit simulations. After selection of a candidate complex, time‐dependent behavior can be modeled at atomic resolution using molecular dynamics (MD) simulations of the protein‐ligand system. MD permits the complex to relax in a solvated environment, exposes flexibility and conformational changes, and can determine which interactions (e.g., hydrogen bonds) are maintained throughout the simulation [14, 15]. Binding free energy estimation methods such as Molecular Mechanics and Poisson Boltzmann Surface Area (MM/PBSA) can be performed on MD trajectories to produce a more precise estimate of affinity [14]. Türkoğlu et al. [16] examined the studied lysozyme–tannic acid system through docking, MD simulation, and SPR validation. The study reported docking binding energies (~−7.5 kcal/mol) and calculated the binding free energy from MD simulations to be −27.6 kcal/mol. The authors noted that hydrophobic contacts and van der Waals forces contributed most to the lysozyme ligand interface [16]. This represents a model which we followed; a comparable methodology, using lysozyme or α‐amylase with HEMA or TEGDMA, can be employed to determine interacting residues and their binding affinities.

Although resin monomer–protein interactions are clinically important, previous computational investigations have been limited in three ways. First, previous studies have been limited to assessing either a single monomer or single protein target, with little direct comparison made between affinities to multiple salivary proteins. Second, density functional theory (DFT), molecular docking, MD, force‐field‐based simulation, and MM/PBSA binding free‐energy analysis have rarely been used collectively in resin monomer studies. Third, no previous investigations have related molecular electronic properties with conformational behavior and thermodynamic binding trends [1, 2].

In order to bridge these limitations, we herein benchmarked three dentally‐relevant resin monomers, HEMA, Bis‐GMA, and TEGDMA, against a wide panel of salivary proteins. Molecular docking was combined with DFT calculations, OPLS‐AAA‐based MD simulations, MM/PBSA binding free‐energy estimation, and per‐residue energy decomposition to relate electronic structure information with conformational mobility, dynamic protein−ligand interactions, and predicted binding energetics across these chemical model systems. This allowed for a cross‐scale interpretation of monomer recognition by salivary proteins.

The present study was designed as a mechanistic comparative investigation of three widely used and clinically relevant dental resin monomers rather than as a virtual‐screening study for the discovery of new monomer candidates. HEMA, Bis‐GMA, and TEGDMA were selected because they are commonly incorporated into dental resin materials and have documented potential for release into the oral environment. Identification and computational screening of novel resin monomers represents an important future extension of this work, in which the present integrated docking, DFT, MD, and binding free‐energy workflow could be applied to prioritize candidate monomers with more favorable predicted biocompatibility profiles.

## 2 Methodology

### 2.1 Selection and Preparation of Dental Resin Monomers and Salivary Proteins

The materials investigated in this study were common dental resin monomers and a large collection of salivary proteins critically important for oral homeostasis, enzymatic function, antimicrobial activity, mineral homeostasis, and pellicle formation on enamel. Materials were selected with the goal of performing a comparative computational analysis of interactions between salivary proteins and clinically relevant monomers that may influence biocompatibility of resin‐based dental materials. HEMA (PubChem CID: 13,360), Bis‐GMA (PubChem CID: 15,284), and TEGDMA (PubChem CID: 7979) were chosen as representative ligands, and their structures were downloaded from PubChem [16] (https://pubchem.ncbi.nlm.nih.gov/, accessed 12th November 2025) as structure data files (SDF). Structures for these three methacrylate‐based monomers were chosen due to their prevalence in resin composites and dental adhesives and previous reports demonstrating these monomers can leach into saliva after incomplete polymerization. Together, these three monomers span a range of molecular sizes, flexibility, and hydrophilicity, permitting comparison of their interaction behavior with salivary proteins. No virtual screening for novel resin monomers was undertaken because the objective of the present study was to characterize the molecular behavior of representative monomers already used in dental resin systems.

Salivary proteins were chosen after thorough consideration of the literature on oral biology, proteomics, and dental materials science. Candidate proteins are proteins that are (1) highly expressed in human saliva; (2) functionally important to oral health, including antimicrobial protection, food digestion, and mineral homeostasis; (3) implicated in acquired enamel pellicle formation; and/or (4) may likely interact with exogenous small molecules in the oral cavity. Selected proteins included enzymatic, antimicrobial, mineral‐binding, immune‐related, carrier, and structural salivary proteins. When available, experimentally resolved three‐dimensional structures were downloaded from the protein data bank. When a suitable experimentally resolved structure was unavailable, high‐confidence predicted structures from AlphaFold were downloaded for each respective salivary protein. Sources of protein structures were noted. Predicted structures were taken with caution as protein flexibility in response to ligand binding or the precise geometry of experimentally resolved binding sites may not be represented in protein predictions. Protein structures were visually inspected to ensure structural completeness. Nonessential heteroatoms, crystallographic water molecules, and cocrystallized ligands were eliminated prior to the computational analysis.

If residues were missing from a protein structure, they were modeled using Modeler 10.8 [17] to generate continuous protein models amenable to docking and MD simulation. Active‐site residues annotated as catalytic or functionally important for enzymatic salivary proteins were identified using PDBsum [18]. For nonenzymatic salivary proteins like carrier proteins, immune‐related proteins, mineral‐binding proteins, and pellicle‐associated proteins, we instead searched for regions annotated as a “binding region.” Selection of catalytic active sites or protein‐ligand binding regions included reported ligand‐binding residues, surface‐accessible cavities, hydrophobic grooves and charged patches, and/or predicted pockets that we anticipated would bind small exogenous molecules like resin monomers. CASTpFold [19] was also used to predict possible binding pockets and surface grooves along with important interacting residues for proteins missing experimental annotation of ligand binding available in PDBsum. We did this in order to consider any likely monomer‐accessible regions and not limit docking to catalytic active sites alone. Salivary proteins used for analysis in this study can be found to be summarized in Table 1.

**TABLE 1:** Annotated active sites or predicted ligand‐binding regions of salivary proteins used for docking with dental resin monomers.

Protein	Major oral function	Active site/predicted ligand‐binding region residues	PDB ID; chain
α‐amylase	Starch digestion, pellicle formation	100, 158, 167, 195, 201, 298, 337	1SMD; A [20]
α‐enolase	Surface adhesion, pellicle	37, 38, 39, 157, 165, 244, 292, 317, 342, 371, 372	2PSN; A [21]
Carbonic anhydrase VI (CA6)	Salivary buffering, enamel protection	72, 73, 111, 113, 138, 173, 176, 200, 202, 203	3FE4; A [22]
Kallikrein‐1	Proteolysis, peptide processing in saliva	57, 94, 95, 99, 102, 193, 195, 227, 228	1SPJ; A [23]
Lysozyme	Bacterial cell wall lysis	35, 53	1LZ1; A [24]
Lactoferrin	Iron sequestration	60, 92, 117, 121, 122, 123, 124, 192, 253, 395, 435, 461, 465, 466, 467, 468, 528, 597	1B0L; A [25]
Lactoperoxidase	Antimicrobial oxidative system	40, 43, 98, 100, 101, 116, 161, 195, 425	2R5L; A [26]
Histatin‐5	Antifungal activity	35, 39, 49, 50, 53	1HST; A [27]
β‐Defensin‐1	Innate immunity	1, 2, 3, 4, 12, 17, 25, 27, 28, 29	1IJV; A [28]
Immunoglobulin A (IgA Fc)	Mucosal immunity	41, 42, 43, 44, 71, 72, 75, 178, 179, 182	1OW0; C [29]
Salivary agglutinin (gp340 fragment)	Bacterial aggregation	71, 72, 73, 74, 94, 95, 97, 98, 99	8J8D; A [30]
Protein S100‐A8	Inflammatory signaling	20, 23, 25, 28, 30, 59, 61, 63, 65, 70	1MR8; A [31]
Protein S100‐A9	Immune modulation	19, 23, 29, 30, 31, 32, 74, 75, 76, 79	7UI5; A [32]
Annexin A1	Anti‐inflammatory response	81, 117, 124, 128, 129, 145, 147, 212, 234, 235, 238, 249	1HM6; A [33]
Annexin A2	Cell–membrane interaction	49, 50, 52, 87, 90, 95, 117, 119, 121, 122, 124, 161, 201, 204, 206, 244, 246, 247, 252, 277, 279, 281, 321	2HYW; A [34]
Statherin	Enamel remineralization	28, 31, 38, 39	AF‐P02808‐F1‍‐‍‍v6; A [35]
Mucin 1	Surface lubrication, pellicle formation	1041, 1042, 1067, 1068, 1095, 1096	2ACM; A [36]
Cystatin C	Tissue protection	34, 40, 44, 70, 93	1G96; A [37]
Thioredoxin	Oxidative stress control	26, 28, 29, 30, 35, 36, 39, 56, 58, 75	1ERT; A [38]
Peroxiredoxin‐1	Reactive oxygen species (ROS) scavenging	55, 128	1QQ2; A [39]
Heat shock protein 70 (HSP70)	Protein protection	137, 148, 385, 386, 412, 417, 506, 507, 512	2KHO; A [40]
Serum albumin (salivary fraction)	Ligand binding	30, 31, 32, 106, 107, 462, 463, 478, 484	1AO6; A [41]
Zinc‐alpha‐2‐glycoprotein (AZGP1)	Small‐molecule and lipid binding in saliva	14, 73, 76, 77, 101, 115, 117, 134, 148, 154, 161	1ZAG; A [42]

*Note:* AF‐P02808‐F1‐v6 represents an AlphaFold‐predicted structure rather than an experimentally resolved PDB structure. All other listed structures were obtained from experimentally available PDB entries where suitable structures were available.

The saliva proteins chosen together make up components of the innate defense system and the oral surface interface that surrounds dental materials. α‐amylase, carbonic anhydrase VI, and lactoperoxidase are digestive, buffering, and antibacterial enzymes. Lysozyme, histatins, defensins, and lactoferrin are proteins involved in the natural defense system [43, 44]. Statherin and proline‐rich proteins are calcium‐binding proteins involved in enamel stabilization and calcium–phosphate equilibrium [45, 46]. Immunoglobulin A (IgA) and salivary agglutinin are immune system‐related proteins that affect bacterial adherence and biofilm accumulation [47, 48]. For this reason alone, these proteins were selected since they are abundant proteins with surface‐active properties that may be important with regard to molecular‐level interactions of leached resin monomers to gain insight into biocompatibility, inhibition, and long‐term cytotoxic effects.

### 2.2 Selection of Reference Ligands for Comparative MD and MM/PBSA Analysis

Reference ligands were used during MD/MM‐PBSA analyses to serve as protein‐specific references with which to better understand the relative stabilities/bindings free‐energy landscapes of the dental resin monomers. Ideally, one could use universal positive/negative controls for all salivary protein targets. Unfortunately, this is impossible, as each protein serves a unique biological role with distinct binding‐site topology and ligand recognition patterns. As such, each reference ligand was chosen with respect to its relevance to the protein system under study. Caffeine and trans‐chalcone were chosen for α‐amylase as previously reported small‐molecule binders/modulators of α‐amylase and served as references for small‐molecule behavior within the enzyme’s catalytic/binding groove. Glucosamine and diacetylchitobiose were chosen for lysozyme, as they are analogs of carbohydrates/substrate fragments of lysozyme carbohydrate substrates and are physiologically relevant to lysozyme’s binding cleft. Ibuprofen and warfarin were chosen for serum albumin as they are established site‐marker ligands for albumin’s drug‐binding sites and are frequently examined for binding within albumin’s major hydrophobic pockets.

Hence, the reference ligands were solely used to put into perspective the predicted MD stability and MM/PBSA binding free energies of HEMA, Bis‐GMA, and TEGDMA in each individual protein system. No assertions of equal biological activity between proteins should be inferred, nor were intentions to use these as absolute benchmarks for strong or weak binding.

### 2.3 Molecular Docking Simulations

Docking simulations were carried out for the selected dental resin monomers HEMA, Bis‐GMA, and TEGDMA, with the selected salivary proteins to predict binding modes and interaction patterns of these compounds in order to determine possible molecular associations with these proteins that could relate to oral biocompatibility. The structures of all the ligands were geometry‐optimized prior to docking to release steric strain and allow the generation of low‐energy conformations favorable for docking. Ligand preparation consisted of an energy minimization using the MM2 force field [13, 49, 50, 51]. MM2 force field optimization is a standard approach used for geometry optimization of small organic molecules to generate low energy conformations by optimizing bond lengths, bond angles, and torsional geometry. Protein–small molecule docking simulations were performed using HADDOCK [52]. HADDOCK has been successfully used for docking proteins to small molecules and allows for flexible refinement of the ligand–protein complexes. Protein–ligand docking simulations were performed using the HADDOCK2.4 Web server/HADDOCK 2.5 backend (accessed on November 2025) via the Utrecht Biomolecular Interaction Web Portal. Structures were prepared for docking by deleting nonessential heteroatoms and crystallographic waters. Cocrystallized ligands were deleted from the proteins unless they were necessary for the definition of the binding site. Docking restraint definitions were based on the catalytic residues, annotated ligand‐binding residues, or predicted surface‐accessible pocket residues (Table 1). The resulting complexes were clustered based on structural similarity and ranked according to the HADDOCK score. The HADDOCK score incorporates van der Waals, electrostatic, desolvation, and restraint‐violation energy terms. The top‐ranked pose/cluster (complex ensemble with the best HADDOCK score) was chosen based on the cluster consistency, predicted binding energy, and visual examination of the occupancy of the binding pocket.

Protein and ligand structures were processed by deleting nonessential heteroatoms, crystal waters, and cocrystallized ligands that did not define the binding site. Polar hydrogens were added, proteins were assigned protonation states at pH 7.4, and ligands were kept with rotatable bonds enabled. Docking restraints included residues in Table 1, which corresponded to catalytic residues, annotated ligand‐binding residues, or predicted surface‐accessible binding pockets. For each protein–ligand pair, the following default HADDOCK2.4 web‐server sampling was performed: 1000 structures were generated in rigid‐body docking, the top 200 structures were refined in semiflexible mode, and the top 200 structures were refined in explicit solvent. Explicit water‐refined models were clustered with the default HADDOCK ligand clustering parameters using the fraction of common contacts and/or ligand RMSD as the criterion. Default ligand clustering cutoffs were set to 0.6 and 1.5 Å. The top‐ranked cluster was chosen by its HADDOCK score, cluster size, predicted binding energy, and visual inspection of occupancy in the binding pocket.

Docking regions were selected to correspond with biologically significant catalytic sites when available, annotated ligand‐binding regions when available, or predicted surface‐accessible pockets generated during protein preparation. Alternatively, docking was centered around annotated or predicted ligand‐binding cavities rather than catalytic active site(s) for carrier/proteins lacking enzymatic function (albumin). For each monomer–protein pair, a series of docking poses were produced. Generated complexes were clustered by structural similarity and ranked using HADDOCK’s scoring function, which takes into account van der Waals interactions, electrostatic interactions, desolvation energy, and restraint violations. Scoring. The predicted binding affinity of highest‐ranking docking complexes was further characterized by predictive estimates of binding free energy and dissociation constants (KD) using the PRODIGY (PROtein binDIng enerGY prediction) server [53]. PRODIGY calculates predicted binding affinity based on interfacial contacts and structural properties unique to each docked complex. Provided a quantitative estimate of predicted binding affinity, HEMA, Bis‐GMA, and TEGDMA could then be qualitatively ranked for each protein in the salivary panel to identify monomers with preferential or diminished affinity. Top‐ranked docking poses were then used for subsequent DFT calculations and MD simulations.

Site‐specific docking was employed, targeting only catalytically or functionally relevant binding pockets to enrich for biologically relevant monomer–protein interactions. Blind docking of monomers to the entire protein surface was not done. Hence, we cannot rule out the existence of additional weak or alternative binding sites outside the targeted regions. This approach was chosen to balance biological relevance with computational efficiency, while future studies may extend the analysis through blind docking or systematic scanning of multiple surface‐accessible pockets.

Docking validation was performed with reference ligands of the selected protein targets to increase confidence in the docking protocol. Validation was only performed when structurally or functionally informative ligand‐bound information was available. Predicted ligand‐binding surfaces were mapped against reported ligand‐binding or active‐site residues from PDBsum/CASTpFold annotations. Acceptable docked poses reasonably occupied the predicted binding pocket and had spatial agreement with the known interacting residues. For these reasons, docking scores and estimated binding energies were used for relative prioritization of monomer–protein complexes instead of relying on them as predictive of absolute predicted binding affinity.

Hydrogen bonds were based on a donor–acceptor distance cutoff of ≤3.5 Å with an appropriate donor–hydrogen–acceptor geometry when possible. Interactions greater than this distance were referred to as polar contacts, proximity contacts, or van der Waals contacts rather than a traditional hydrogen bond.

### 2.4 DFT Calculations

Docking and MD simulations require calibration against experiments and quantum chemistry calculations. DFT calculations on the dental resin monomers were performed using a custom‐developed Python workflow [54]. Bond geometry optimizations were completed for all molecular structures using the PBE0/6‐31G(d) level of theory. Default tight convergence parameters were used (energy convergence tolerance of 10^−6^ Hartree and maximum force tolerance of 10^−3 ^Hartree/Bohr) to locate energetically favorable geometries. Molecular orbital (MO) eigenvalues were collected after bond optimizations using single‐point calculations at the same level of theory. The highest occupied molecular orbital (HOMO) and lowest unoccupied molecular orbital (LUMO) energies were extracted automatically by the workflow script. The energy gap between the HOMO and LUMO energies (Δ*E*) was calculated and can be used as a reference for molecular stability, electronic excitability, and chemical reactivity. Standard visualization tools were used to depict frontier MO distributions with an isovalue of 0.02 a.u. to qualitatively identify electron‐rich regions and reactive sites. Quantum chemistry software used and the corresponding input files are available in the Supporting Information data.

### 2.5 MD Simulations

MD simulations were carried out to explore the dynamic properties, stability, and if complexes remained intact overtime of the docked complexes created between each dental resin monomer, HEMA, Bis‐GMA, and TEGDMA, and selected salivary proteins. All MD simulations were run through GROMACS 2025.4 [55]. The protein–ligand systems were described using theoptimized potentials for liquid simulations all‐atom force field (OPLS‐AA/L) [56, 57]. Ligand topology and force‐field parameters were generated consistently using the OPLS‐AA/L force field, and partial charges were calculated prior to system setup.

The protein–ligand complex was positioned in a cubic simulation box, and the solute was at least 1 nm away from the edge of the box in all directions to prevent interactions with its periodic image. Systems were solvated with the Simple Point Charge Extended (SPC/E) water model, neutralized with counterions, and then ions were added to mimic physiological concentrations [14, 58].

Following system assembly, energy minimization was performed using the steepest descent method until reaching a convergence threshold. Energy‐minimized systems were then subjected to 2 stages of restrained equilibration under NVT (310 K) followed by NPT (1 bar) to reach equilibrium temperature and pressure/density [59, 60].

Long‐range electrostatic interactions were calculated with the particle mesh Ewald method. Short‐range nonbonded interactions were calculated with a cut‐off distance. All hydrogen‐containing bonds were constrained, which enabled the use of a 2 fs integration timestep. Production MD simulations were carried out for 100 ns on each monomer–protein and reference ligand–protein complex. All systems received the same MD treatment so that the results could be interpreted comparatively.

Trajectory frames were recorded throughout the 100 ns production simulations and analyzed after trajectory processing. The calculated parameters included root‐mean‐square deviation (RMSD), root‐mean‐square fluctuation (RMSF), radius of gyration (RoG), solvent‐accessible surface area (SASA), hydrogen‐bond occupancy, ligand–protein center‐of‐mass distance, and interaction energies in Å^2^ . All simulations were run on a desktop workstation with an Intel Core i7‐14700F @3.2 GHz processor, 32 GB of DDR5 memory, 1 TB of SSD, and an NVIDIA GeForce RTX 50 GPU.

Only one 100 ns production MD trajectory was performed per protein–ligand complex. Replicate simulations with varied initial velocity seeds were not performed. The reported RMSD, RMSF, RoG, SASA, hydrogen bond, and ligand‐distance profiles should be considered descriptive and trend‐oriented observations made from a single trajectory. They should not be interpreted as statistically reproducible differences between ligands. Inferential statistical analyses cannot be made about complex stability/convergence based on these data.

### 2.6 MM/PBSA Calculations

Binding free energies of the selected HEMA, Bis‐GMA, and TEGDMA–salivary protein complexes were calculated using the MM/PBSA method, as implemented in gmx_ MM/190.

PBSA version 1.6.4 [61]. MM/PBSA calculations were carried out on snapshots taken from the equilibrated portion of the 100 ns MD trajectories. The initial equilibration portion of each production trajectory was discarded to avoid biasing our calculations with structures that have not completely relaxed, and representative frames were pulled from the equilibrated region of the trajectory for use in binding free‐energy estimation. The total binding free energy (Δ*G* binding) is computed as the sum of molecular mechanics energy terms, which include van der Waals and electrostatic contributions, in addition to polar and nonpolar solvation free energy terms. The polar solvation contribution was computed using the Poisson–Boltzmann model. Nonpolar solvation free energy was calculated from the SASA.

Entropic contributions were not computed due to their computational expense, and large deviations were observed in systems of this comparative size. As such, MM/PBSA values were considered estimates of relative binding free energy but not direct comparison to experimental binding affinities. Per‐residue energy decomposition was used to explore favorable and unfavorable energetic contributions within the sampled complexes. These contributions were interpreted descriptively and were not used independently to establish binding affinity, ligand residence time, or simulation convergence.

MM/PBSA binding energies are presented as the average ± standard deviation from the frames analyzed from a single 100 ns production trajectory for each complex. Entropic terms were not included in these calculations. The standard deviations reflect differences between frames sampled within a given trajectory and do not refer to the reproducibility between independent runs. Statistical significance was not inferred from the overlap or lack thereof between these standard deviations. No inferential statistics were made between ligands. The means provided are intended as exploratory estimates that are conditional on the conformations sampled and the settings used to calculate them and may not have been converged.

## 3 Results

### 3.1 Molecular Docking Overview and Predictive Binding Affinity Trends

Bis‐GMA, HEMA, and TEGDMA all exhibited distinct but similar binding profiles with saliva proteins. Binding profiles appeared to be dependent on both the size and flexibility of each molecule as well as the functional group chemistry each molecule possessed. The top five docking poses scored by both the HADDOCK score and predicted binding energy for each protein–monomer combination are shown in Table 2. All docking data can be found in Data S1. α‐amylase, lysozyme, and serum albumin were found within the top five binding partners for each monomer, indicating that each monomer has favorable predicted interactions with possible leachable dental resin compounds found in saliva. Bis‐GMA possessed the strongest mean docking score of the three molecules. Predicted binding energies as low as −10.06 kcal/mol were calculated for the Bis‐GMA–α‐amylase complex. This was followed by TEGDMA (the lowest docking score of −8.05 kcal/mol) and HEMA (the lowest docking score of −6.74 kcal/mol). All docking RMSD values were low between each protein–ligand complex, indicating that the poses within each docking run clustered tightly around each other. This suggests that there may only be one preferred docked conformation that is maintained across multiple runs. Regardless, this value does not portend any biological relevance as to the stability of each complex, as all complexes underwent MD simulation to evaluate dynamic stability. Energy contribution decomposition also indicated that van der Waals and electrostatic interactions were the leading source of binding for Bis‐GMA and TEGDMA. Hydrophobic, polar, and hydrogen‐bond‐donating/‐accepting groups played a larger role in the binding of HEMA.

**TABLE 2:** Top five docking complexes between dental resin monomers and salivary proteins based on HADDOCK scores and predicted binding energy (full docking dataset provided in Data S1).

Monomer‐salivary protein complex	HADDOCK‍‍score, a.u.	Binding energy, kcal/mol	Van der‍ waals energy	Electrostatic energy	Desolvation energy	RMSD
Bis‐GMA_α‐amylase	−52.5 ± 1.0	−10.06	−39.4 ± 0.5	−43.9 ± 4.4	−14.8 ± 0.4	0.1 ± 0.1
Bis‐GMA_albumin	−40.2 ± 2.0	−9.29	−33.2 ± 1.1	−75.2 ± 4.5	−2.0 ± 0.4	0.3 ± 0.1
Bis‐GMA_annexin A1	−19.4 ± 4.1	−8.95	−25.7 ± 1.2	−62.2 ± 5.6	−1.6 ± 0.2	0.2 ± 0.0
Bis‐GMA_mucin 1	−49.0 ± 1.8	−8.95	−43.6 ± 2.3	−1.4 ± 1.7	−14.0 ± 0.7	0.7 ± 0.1
Bis‐GMA_lysozyme	−39.7 ± 1.0	−8.70	−31.4 ± 1.0	−48.5 ± 0.5	−3.5 ± 0.4	0.1 ± 0.0
HEMA_AZGP1	−23.4 ± 0.1	−6.74	−14.5 ± 0.1	−33.3 ± 1.1	−5.6 ± 0.1	0.1 ± 0.1
HEMA_lysozyme	−18.8 ± 0.1	−6.66	−14.1 ± 0.1	−58.1 ± 0.9	1.1 ± 0.1	0.2 ± 0.0
HEMA_α‐amylase	−7.7 ± 1.3	−6.48	−13.1 ± 1.1	−15.2 ± 0.7	−0.6 ± 0.2	0.2 ± 0.0
HEMA_CA6	−16.9 ± 0.5	−6.46	−17.1 ± 0.5	−35.7 ± 1.9	−2.1 ± 0.5	0.1 ± 0.0
HEMA_albumin	−21.7 ± 0.1	−6.37	−17.8 ± 0.1	−29.8 ± 1.9	−0.9 ± 0.2	0.2 ± 0.0
TEGDMA_lysozyme	−34.0 ± 0.6	−8.05	−22.3 ± 0.2	−88.9 ± 4.3	−2.9 ± 0.6	0.1 ± 0.0
TEGDMA_AZGP1	−34.4 ± 0.3	−7.79	−20.6 ± 0.6	−43.4 ± 1.1	−9.5 ± 0.5	0.1 ± 0.1
TEGDMA_α‐enolase	−29.2 ± 0.3	−7.45	−23.2 ± 0.2	−62.0 ± 3.9	0.1 ± 0.3	0.1 ± 0.0
TEGDMA_albumin	−30.1 ± 0.3	−7.41	−20.9 ± 0.2	−48.5 ± 3.4	−4.4 ± 0.2	0.1 ± 0.0
TEGDMA_α‐amylase	−26.9 ± 1.4	−7.35	−23.6 ± 0.6	−46.1 ± 2.8	−3.7 ± 0.8	0.1 ± 0.0

The 2D interaction maps revealed that the monomer–protein complexes were stabilized by different combinations of hydrogen bonding, close polar contacts, van der Waals interactions, and π‐related hydrophobic interactions depending on monomer size, polarity, and structural flexibility. As depicted in Figure 1A–C, Bis‐GMA formed extensive interacting networks within α‐ amylase, lysozyme, and albumin binding sites due to its bulky aromatic core and its numerous ester functional groups. In the Bis‐GMA–α‐amylase complex (Figure 1A), the ligand formed a close polar contact with Lys200 (3.87 Å) and a carbon–hydrogen/proximity contact with Tyr151 (5.61 Å). Van der Waals contacts with neighboring residues (Ile235 and Leu162) and π–alkyl and π–sigma interactions with aromatic residues Trp_58_ and Tyr_62_ further stabilized this complex, explaining its favorable HADDOCK score (−52.5) and predicted binding energy (−10.06 kcal/mol). In the Bis‐GMA–lysozyme complex (Figure 1B), Bis‐GMA adopted an extended confirmation within a shallow binding pocket and formed a carbon–hydrogen/proximity contact with Glu_35_ (5.57 Å). Van der Waals contacts with Asn_44_, Ala_111_, and Val_110_ as well as aromatic π–alkyl contacts with Tyr_63_ and Trp_109_ stabilized this complex, explaining its favorable predicted binding energy (−8.70 kcal/mol). In the Bis‐GMA–albumin complex (Figure 1C), Bis‐GMA formed a close polar contact with Val462 (4.28 Å) and carbon–hydrogen/proximity contacts with Asp107 (4.54 Å) along with extensive van der Waals contacts along its extended ligand scaffold. π–alkyl interactions with Lys466 and neighboring hydrophobic residues also stabilized this complex, as depicted in its electrostatic contribution (−75.2 kcal/mol) in Table 2.

**FIGURE 1:**
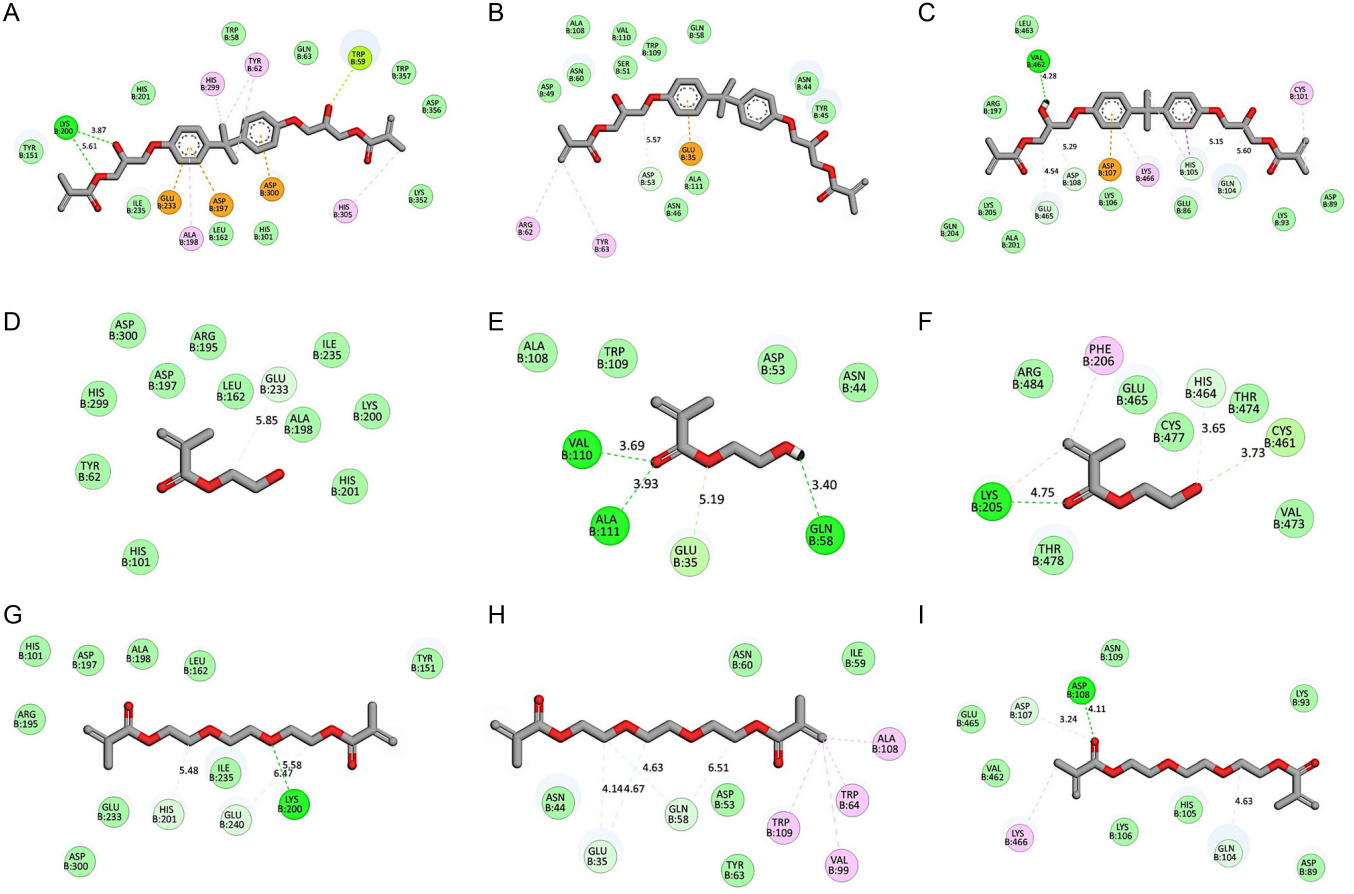
Two‐dimensional interaction maps of dental resin monomers with selected salivary protein binding sites. (A) Bis‐GMA–α‐amylase complex. (B) Bis‐GMA–lysozyme complex. (C) Bis‐GMA–albumin complex. (D) HEMA–α‐amylase complex. (E) HEMA–lysozyme complex. (F) HEMA–albumin complex. (G) TEGDMA–α‐amylase complex. (H) TEGDMA–lysozyme complex. (I) TEGDMA–albumin complex. Hydrogen bonds, carbon–hydrogen bonds, van der Waals interactions, Pi–alkyl interactions, and Pi–sigma interactions are indicated by the corresponding color coding.

HEMA formed fewer and less extensive contacts with the tested proteins (Figure 1D–F), presumably because of the decreased molecular size and higher hydrophilicity. In the HEMA–α‐amylase complex (Figure 1D), HEMA formed a carbon–hydrogen/proximity interaction with Ala198 (5.85 Å). Weak van der Waals contacts with neighboring residues included Asp197, Glu233, and Lys200. Because of its low number of interacting surface area residues, its predicted binding energy was less favorable (−6.48 kcal/mol). Hydrogen bond interactions in the HEMA–lysozyme complex (Figure 1E) included a conventional hydrogen bond with Gln58 (3.40 Å) as well as close polar interactions with Val110 (3.69 Å) and Ala111 (3.93 Å). While these residues were able to confer additional interactions to bind to lysozyme, they did not meet strict geometric definitions to be considered conventional hydrogen bonds. Close polar interactions were again noted between HEMA and albumin at Cys477 (3.65 Å) and Cys461 (3.73 Å) (Figure 1F). Lys205 was noted to form a carbon–hydrogen/proximity interaction with HEMA at 4.75 Å. Weak van der Waals contacts with Glu465 and Val473 also aided in stabilizing the ligand in the albumin‐binding pocket.

TEGDMA fell in between Bis‐GMA and HEMA in regard to binding and showed conformational freedom. However, TEGDMA contained multiple ester groups (>1) (Figure 1G–I). While in complex with α‐amylase (Figure 1G), the ligand engaged in long‐range polar/proximity contacts with Lys200 (5.48 and 6.47 Å) and therefore did not make a conventional hydrogen bond. This was made possible by van der Waals interactions with Ile235, Leu162, and Ala198 and agreed with its predicted binding energy of −7.35 kcal/mol. When bound to lysozyme, TEGDMA established polar/proximity contacts with Asn_44_ (4.14–4.67 Å) and a carbon–hydrogen/proximity contact with Gln_58_ (6.51 Å) (Figure 1H). TEGDMA formed π–alkyl interactions with Trp109 and Val_99_, which strengthened the binding and explained the high contribution from electrostatics (−88.9 kcal/mol) and agreed with the predicted binding energy of −8.05 kcal/mol. The TEGDMA–albumin complex exhibited one conventional hydrogen bond/close polar contact with Asp108 (3.24 Å) and a polar contact at 4.11 Å (Figure 1I). Other residues that formed van der Waals interactions include Glu465, Val462, and His105. Albumin formed a π–alkyl interaction with the ligand through Lys466, which further stabilized the complex and agreed with its predicted binding energy of −7.41 kcal/mol.

The docking poses of Bis‐GMA, HEMA, and TEGDMA depicted in Figure 2 illustrate how these molecules can structurally fill the ligand‐binding domains (LBDs) of α‐amylase, lysozyme, and serum albumin. Docking simulations placed monomers from each complex into surface‐accessible cavities/clefts present on all three proteins. This observation coincides with predictions that these monomers would be highly exposed, lending themselves to interact with proteins in saliva during physiological conditions. While the three monomers differ in size and structural flexibility, all three assumed protein‐complementary shapes that fit snugly into their respective protein binding pockets. In α‐amylase’ LBD (Figure 2A), Bis‐GMA became deeply inserted into a long crevice on the enzyme surface and rested flat against the long axis of the pocket while maintaining an extended conformation. The aromatic rings aligned themselves near hydrophobic surfaces within the cavity, and the ester backbone was nestled into curves of the interior surfaces that make up the binding pocket. Such structural stability upon binding of Bis‐GMA to α‐amylase could be attributed to this insertion into a binding pocket as well as the favorable orientations gained throughout the docking process. Bis‐GMA also filled a binding pocket on lysozyme (Figure 2B); however, this cavity was more shallow than that of α‐amylase. The pocket was wider, and Bis‐GMA comfortably sat in the surface pocket with its rigid aromatic core spanning across the opening of the cavity, while its flexible alkyl tails lounged in adjacent subpockets. When Bis‐GMA was docked into albumin (Figure 2C), it filled a large hydrophobic cleft between domains. Bis‐GMA bent when placed into albumin’s cavity and contacted several subdomains of albumin’s LBD, which correlates with albumins' known ligand‐binding promiscuity.

**FIGURE 2:**
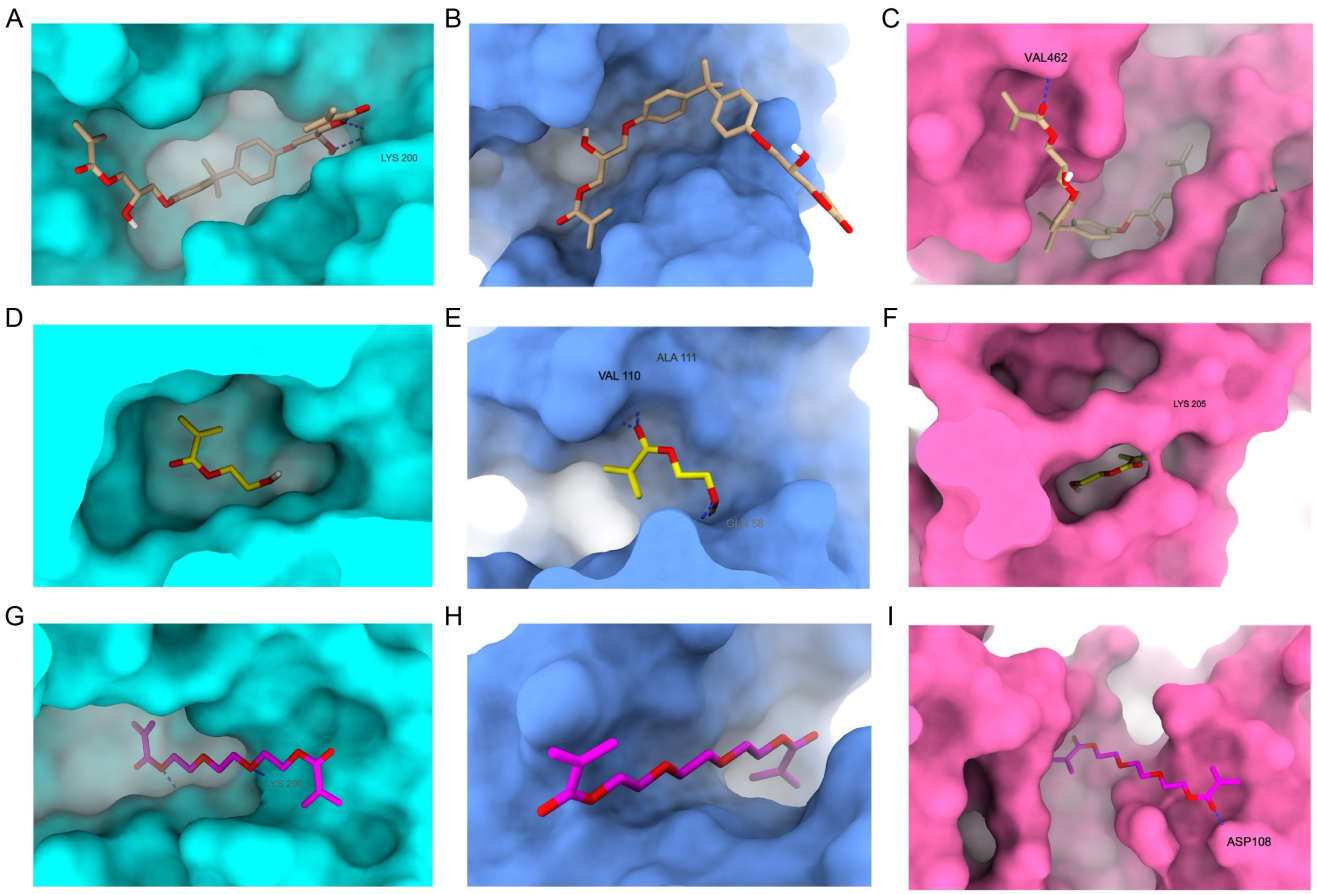
Three‐dimensional best binding poses of dental resin monomers within selected protein binding cavities. (A) Bis‐GMA–α‐amylase complex. (B) Bis‐GMA–lysozyme complex. (C) Bis‐GMA–albumin complex. (D) HEMA–α‐amylase complex. (E) HEMA–lysozyme complex. (F) HEMA–albumin complex. (G) TEGDMA–α‐amylase complex. (H) TEGDMA–lysozyme complex. (I) TEGDMA–albumin complex.

HEMA bound as anticipated due to its small size and polarity. Buried closer to the opening of a pocket on α‐amylase versus deep within the cavity (Figure 2D), HEMA and α‐amylase showed poor steric complementarity with the ligand being tightly curled up into a ball, taking up little space in the binding site. Steric complementarity, improved for lysozyme, where HEMA was buried in a small pocket and oriented such that both the hydroxyl and ester groups pointed toward the pocket interior (Figure 2E). It can be deduced that lysozyme more readily fits small polar monomers due to this. Buried in a small crevice toward the center of the binding pocket but also taking up very little space on albumin (Figure 2F), these features align with albumin’s ability to bind small endogenous and exogenous ligands. As anticipated due to its flexible linear geometry, TEGDMA fell in between the other molecules with regard to the binding properties with all proteins. Buried in α‐amylase such that it stretched throughout a majority of the binding groove in a stretched‐out chain‐like manner (Figure 2G), TEGDMA aligned along the outside edge of the pocket such that its ester groups pointed toward the pocket interior while still allowing for conformational flexibility. Resting halfway inside a cavity on the protein surface for lysozyme with one end in the pocket and the other end remaining solvent‐exposed (Figure 2H), TEGDMA also appeared to have an intermediate burial depth. Lastly, TEGDMA was comfortably nestled in a wide binding cleft on albumin and curved along the pocket, contouring itself to the shape of the binding site and maximizing surface area within the albumin LBD (Figure 2I).

Additional information about monomer–protein recognition aside from binding energies and spatial poses was derived by calculating the frequency at which each type of intermolecular atomic contact occurred for all top‐scoring docking complexes (Table 3). CC and CO contacts were seen most frequently for all monomer–salivary protein pairs, suggesting that hydrophobic and polar interactions involving ester/hydroxyl oxygen atoms comprise the majority of contacts within each complex.

**TABLE 3:** Distribution of intermolecular atomic contact types in the top monomer–salivary protein docking complexes (full contact analysis provided in Data S2).

Monomer‐salivary protein complex	CC	CO	CN	CX	OO	OX	NO
Bis‐GMA_α‐amylase	3040	1521	848	2	194	0	182
Bis‐GMA_albumin	2191	1379	670	68	194	18	177
Bis‐GMA_annexin A1	2183	1200	603	23	161	4	147
Bis‐GMA_mucin 1	2565	1149	533	6	121	0	130
Bis‐GMA_lysozyme	2046	1286	674	0	173	1	156
HEMA_AZGP1	841	585	155	3	78	0	85
HEMA_lysozyme	596	472	184	0	99	1	94
HEMA_α‐amylase	810	601	232	2	106	2	110
HEMA_CA6	666	481	154	5	84	3	73
HEMA_albumin	619	486	198	14	88	7	89
TEGDMA_Lysozyme	1287	979	389	14	186	2	172
TEGDMA_AZGP1	1487	953	316	3	143	0	127
TEGDMA_α‐enolase	1100	904	389	0	191	0	163
TEGDMA_albumin	918	717	283	29	147	12	119
TEGDMA_α‐amylase	1350	936	360	1	167	2	147

The Bis‐GMA complexes were observed to form drastically greater CC contacts than other types of contacts. Counts were over 2000 for all protein complexes and reached up to 3040 CC contacts for Bis‐GMA–α‐amyelase. This is likely due to Bis‐GMA’s bulkiness and aromatic nature, which enables vast regions of hydrophobic surface complementarity between Bis‐GMA and binding partner cavity surfaces. Very high counts of CO and CN contacts also serve to emphasize Bis‐GMA’s propensity to have its ester oxygens involved in polar interactions with protein residues at a high frequency. On another note, Bis‐GMA complexes formed with α‐amylase, albumin, and lysozyme had unusually high OO and NO contacts as well. This exemplifies that even interactions able to hydrogen‐bond with heteroatoms play a role in complex stabilization. Significantly fewer atomic contacts of all types were formed with HEMA complexes, with contact numbers approximately an order of magnitude lower than Bis‐GMA. This trend correlates with HEMA’s smaller size and surface area. CC and CO interactions were predominant in this dataset as well; however, they occurred at approximately 1/3–1/4 the frequency of CC and CO interactions in Bis‐GMA. The distribution of CN, OO, and NO contacts was relatively similar amongst all HEMA complexes, namely, the α‐amyelase and lysozyme complexes. This conveys that while HEMA forms fewer total contacts with proteins than Bis‐GMA, a larger proportion of contacts occur with heteroatoms.

TEGDMA had intermediate contact profiles between Bis‐GMA and HEMA. The number of CC contacts ranged from ~900 to ~1500 between proteins. This indicated that TEGDMA’s flexible aliphatic backbone enabled hydrophobic interactions with proteins with long linear binding pockets. Compared to HEMA, TEGDMA made significantly more CO and CN contacts because TEGDMA has multiple ester groups branching off the backbone. Between proteins complexed with TEGDMA, lysozyme and α‐amylase had some of the highest amounts of total contacts, possibly due to their higher docking scores and ability to structurally adapt to flexible ligands. Similarly, α‐amylase, lysozyme, and albumin consistently had the highest number of total atomic contacts among all three monomers, confirming previous predictions that these proteins would be preferential salivary protein binders. Albumin had higher instances of CX and OX contacts because albumin’s binding pockets are more chemically diverse and permissive. Lysozyme kept consistent ratios of hydrophobic and polar contacts.

### 3.2 DFT Analysis of Electronic Properties of Dental Resin Monomers

DFT was used to better understand the native electronic structure of Bis‐GMA, HEMA, and TEGDMA by focusing on frontier MO distribution, energy gaps, and dipole moments. FMO distribution can help us understand the chemical reactivity and polarity of molecules, while orbital energies hint at the inherent stability/reactivity of the monomers. Dipole moments were also calculated to predict how strongly the monomers will interact with biomolecules via noncovalent forces. The distribution of the HOMO and LUMO is displayed in Figure 3, while the orbital energies and dipole moments are reported in Table 4. For Bis‐GMA, most of the electron density from the HOMO was localized on the aromatic rings and neighboring ether groups, showing that the π‐dense regions serve as electron donors (Figure 3A). On the other hand, LUMO density was focused on ester groups and parts of the methacrylate groups, showing these electron acceptor regions. Bis‐GMA had the largest HOMO–LUMO gap of the three monomers tested (8.88 eV), implying inherent stability and lower chemical reactivity. Bis‐GMA also had a relatively small dipole moment (1.60 D), suggesting the molecule is only moderately polar, likely due to its large, rigid structure and predominance of hydrophobic aromatic rings.

**FIGURE 3:**
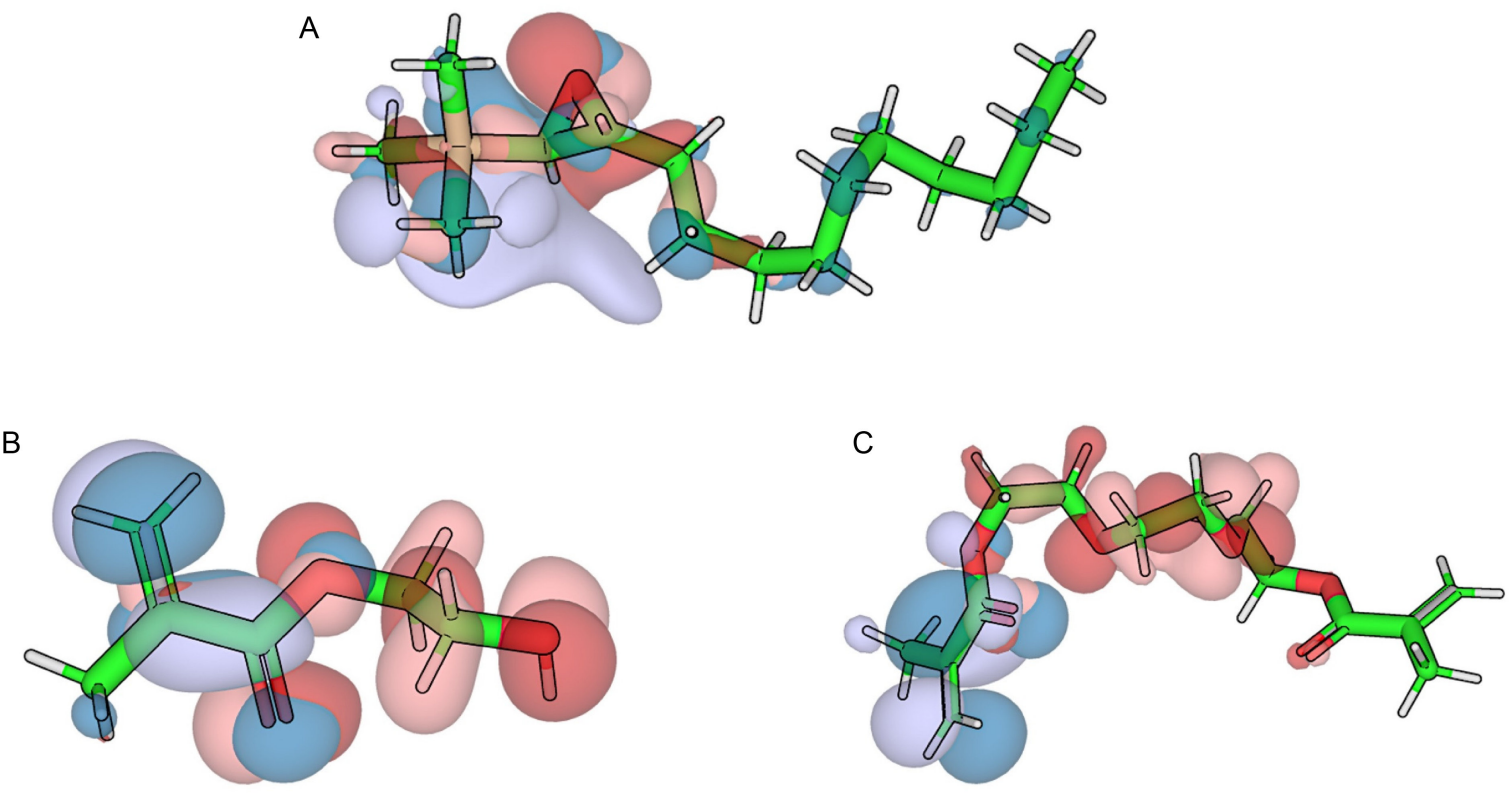
Frontier molecular orbital distributions of dental resin monomers: (A) Bis‐GMA, (B) HEMA, and (C) TEGDMA. Blue surfaces indicate HOMO localization, whereas red surfaces correspond to LUMO regions.

**TABLE 4:** Frontier molecular orbital energies and dipole moments of dental resin monomers obtained from DFT calculations.

Molecule	HOMO, eV	LUMO, eV	Gap, eV	Dipole, D
Bis‐GMA	−6.80	2.08	8.88	1.60
HEMA	−7.44	−1.28	6.16	1.65
TEGDMA	−6.65	−1.08	5.56	3.88

In contrast, HEMA had quite different electronic properties. Its HOMO was located on the hydroxyl and neighboring carbon–carbon double bond, whereas its LUMO was distributed primarily on the ester carbonyl group (Figure 3B). This separation between local electron‐donor and electron‐acceptor regions gave this molecule a distinct polar character, which was also evident by a significantly smaller HOMO–LUMO gap (6.16 eV) than Bis‐GMA, implying that it was electronically softer and hence more reactive toward charge‐ and hydrogen‐bond‐mediated interactions. The computed dipole moment (1.65 D) supported this notion of polarity, which correlates well with its hydrophilicity and the observed preferential interaction with polar protein residues. TEGDMA had the smallest HOMO–LUMO gap (5.56 eV), implying that it is the electronically softest and hence the most reactive monomer (Figure 3C). Its HOMO was delocalized over the aliphatic backbone and ester groups, while the LUMO was most strongly situated on the terminal ester functions, highlighting these motifs as major points of electron acceptance. Interestingly, TEGDMA also had the largest dipole moment (3.88 D), much greater than Bis‐GMA and HEMA, implying significant charge separation. This increased polarity, along with its conformational flexibility, may allow this molecule to better contour electronically to the protein microenvironment. Thus, these electronic descriptors were taken as supplementary to docking contact maps and MD stability measures, not as inherent chemical characteristics, to rationalize effects of molecular size, polarity, electronic softness, and conformational flexibility on monomer–protein interactions.

### 3.3 MD Simulation Results

Several structural descriptors were calculated, including RMSD, RMSF, RoG, SASA, distance of the ligand from the active site, and hydrogen bonding interactions (Figure 4). Since only one simulation was run per complex, results from MD simulations are reported as qualitative and comparative trends calculated from single trajectories. Trends in RMSD, RMSF, RoG, SASA, ligand‐distance, and hydrogen‐bond counts were assessed together to report on relative trends in dynamic behavior. However, the differences should not be interpreted as statistically valid differences between ligands. The MD trajectories were analyzed by visual inspection of time‐series profiles in RMSD, RMSF, RoG, SASA, ligand–protein distance, and hydrogen bond counts. No raw trajectory files were available to extract additional statistical metrics, and therefore, only qualitative analysis was used to interpret the MD results. As such, no quantitative mean ± SD values will be reported for these metrics. Simulations of apo‐lysozyme and apo‐albumin were also analyzed with the described method (Figures S1 and S2) to determine if protein‐specific effects were observed or if the described trends were consistent across salivary and serum proteins. Analysis of RMSD (Figure 4A) showed distinguishable differences between the stability of each complex. The α‐amylase–Bis‐GMA complex maintained the lowest RMSD trajectory, remaining below ~1 Å for the entirety of the simulation. This suggests that there is very little change to the conformation of the protein throughout the simulation and that the protein–ligand complex does not deviate far from the docked conformation. TEGDMA also behaved in a stable manner, with RMSD fluctuations within ~1–2 Å. In contrast, the HEMA complex demonstrated large fluctuations in the RMSD, with numerous spikes above 4 Å and peak deviations near 7–8 Å. These fluctuations suggest that the protein–ligand complex is more flexible and may undergo local structural changes when compared to Bis‐GMA, but not necessarily that the protein is fully destabilized. The protein remaining compact is supported by the RoG and SASA profiles, which did not demonstrate large fluctuations. Of the reference ligands, caffeine showed moderate fluctuations, while trans‐chalcone showed larger fluctuations that were comparable to HEMA, specifically toward the middle to end of the simulation. Together, these results suggest that Bis‐GMA forms the most trend‐level conformational stability with α‐amylase.

**FIGURE 4:**
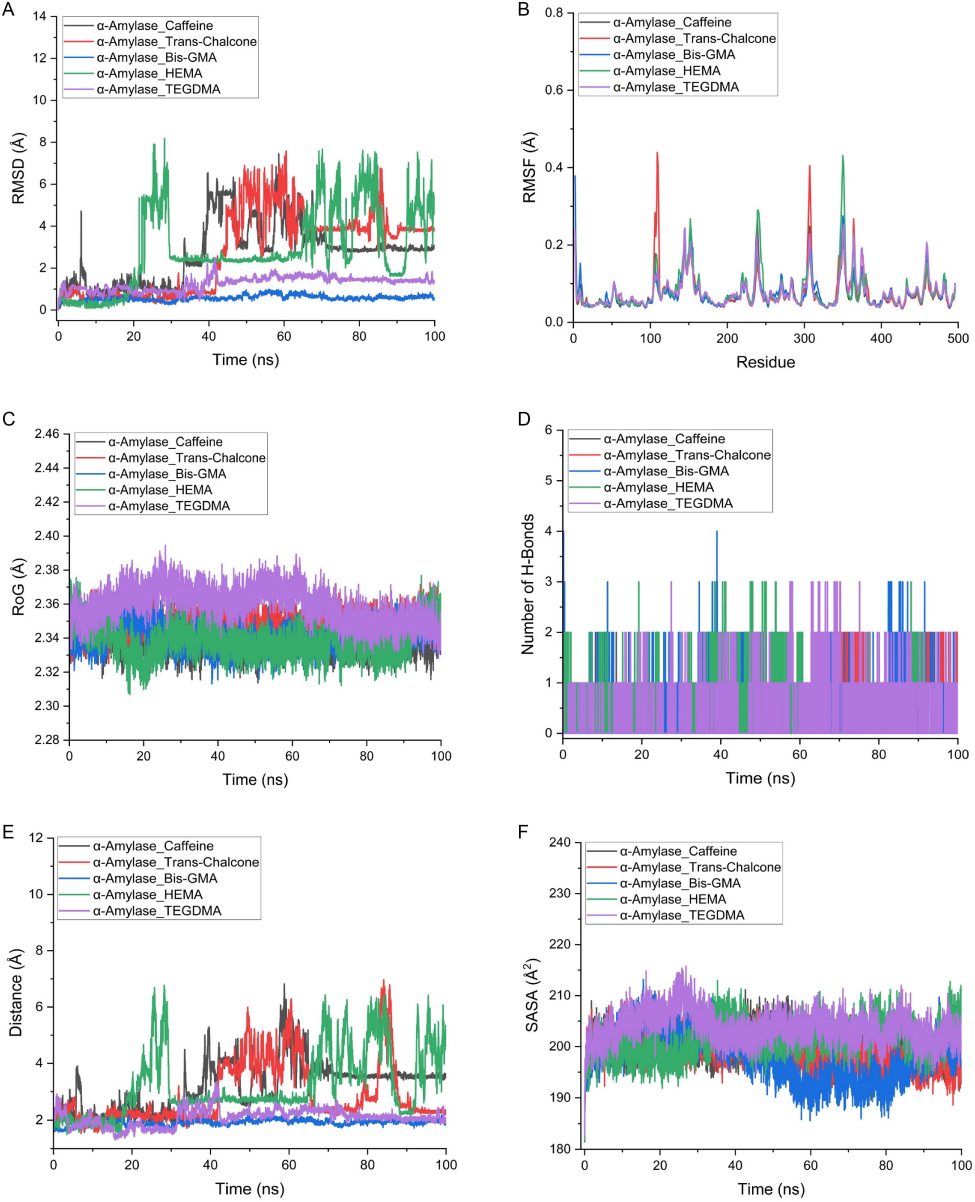
MD simulation results for α‐amylase in complex with dental resin monomers (Bis‐GMA, HEMA, and TEGDMA) and reference ligands (caffeine and trans‐chalcone) over 100 ns of simulation. (A) Root‐mean‐square deviation (RMSD), reflecting the overall conformational stability of the protein–ligand complexes. (B) Root mean square fluctuation (RMSF), providing residue‐level insights into backbone flexibility. (C) Radius of gyration (RoG), indicating protein compactness. (D) Number of hydrogen bonds, reflecting the persistence of polar contacts. (E) Ligand–protein center‐of‐mass distance, illustrating ligand retention within the binding cavity. (F) Solvent‐accessible surface area (SASA), showing changes in surface exposure and solvation upon ligand binding. The ligand–protein distance temporarily increased for HEMA, trans‐chalcone, and caffeine. This suggests some rearrangement within the binding region. It may also indicate partial displacement of the ligands. Longer MD simulations are needed to study this further. Replicate simulations are also required. These studies can show whether the ligands reach more stable conformations. They can also show whether the ligands continue to move within the binding pocket.

HEMA and trans‐chalcone showed larger RMSD changes. Caffeine also showed some fluctuations. This suggests that these complexes adopted different conformations during the 100 ns simulation. However, only one 100 ns simulation was performed for each complex. Therefore, it is not possible to know whether a more stable conformation would appear over a longer simulation time. These changes are better described as conformational flexibility rather than clear instability.

The same hierarchy was seen for simulations with lysozyme (Figure S1A). The complex with glucosamine spontaneously bound was particularly stable (RMSD < ~0.5 Å) and thus acted as a positive control. Bis‐GMA and diacetylchitobiose demonstrated low deviations (~1–2 Å). By comparison, HEMA and TEGDMA experienced greater oscillations of up to ~4–5 Å at different time points. These deviations demonstrate greater structural movement, resulting in weaker stabilization than Bis‐GMA. Albumin simulations (Figure S2A) overall experienced lower RMSD values (<2 Å), highlighting the stability of this transport protein. Both warfarin and ibuprofen demonstrated stable trajectories, while Bis‐GMA moderately equilibrates after initial rebalancing. TEGDMA experiences spikes reaching up to ~2.5–3 Å but later equilibrates, whereas HEMA stays around ~1.5 Å.

The root mean square fluctuation (RMSF) of individual residues (Figure 4B) was similar in each system, suggesting secondary structural features of the enzyme remained unperturbed after ligand binding. Backbone fluctuations of most residues were small (<0.2 Å), and regions with higher flexibility were typically loop regions rather than catalytic portions of the protein. Bis‐GMA and TEGDMA formed complexes with slightly smaller fluctuations in various regions compared to HEMA and trans‐chalcone, indicating marginally better stabilization of backbone movements. Spikes corresponding to flexible loops can be attributed to the protein movement and do not necessarily imply destabilization by the ligand. The RMSF of lysozyme for all ligands was highly overlapped (Figure S1B). Spikes around residue at ~45 and ~70 can be attributed to a flexible loop region. Monomer binding does not significantly alter backbone fluctuations. RMSF values of albumin were higher overall when compared to the enzyme (Figure S2B), likely due to domain‐level motions within this large carrier protein. The trajectories overlapped with each other, indicating that binding did not significantly perturb overall backbone fluctuations. RoG plots also indicate that the overall structure of the complex remained stable. RoG values of all trajectories were tightly grouped between ~2.32–2.40 Å (Figure 4C), indicating that proteins maintained globular structure. Bis‐GMA complex had one of the narrowest distributions, which correlates well with increased stability in folding. Though TEGDMA showed slightly higher RoG values, the increase was marginal and did not suggest unfolding. A similar trend was observed with HEMA, though it produced slightly higher fluctuations. None of the ligands led to global unfolding of the protein. Lysozyme complexes also maintained small RoG values (~1.36–1.42 Å; Figure S1C) similar to the folded protein and had only marginal fluctuations with HEMA and TEGDMA. Albumin trajectories were tightly grouped around ~2.65–2.75 Å (Figure S2C). There was a transient compaction in the Bis‐GMA complex during the initial 5 ns of simulation, which did not last for long, indicating quick equilibration.

Hydrogen bond analysis (Figure 4D) gave additional evidence for the durability of polar contacts. Hydrogen bonds formed in the MD trajectories were tallied with a strict geometric definition33 and are thus reported only as evidence for sustained polar stabilization and not explicit demonstration of strong predictive binding affinity. TEGDMA made the greatest number of hydrogen bonds overall, often sustaining between 1 and 3 contacts throughout the trajectory, reflecting how its multiple ester groups contribute to polar complementarity. Bis‐GMA maintained occasional hydrogen bonds despite its bulkier, more hydrophobic nature, supplementing favorable van der Waals interactions. Hydrogen bonding was moderate for HEMA, though these interactions were temporally less consistent, befitting its higher degree of conformational freedom. Reference ligands maintained comparatively fewer persistent hydrogen bonds, indicating weaker polar stabilization effects. Hydrogen bonds formed with lysozyme (Figure S1D) were most prevalent with diacetylchitobiose (often 3–6), reflecting its polysaccharide‐like makeup, followed by moderate interactions with Bis‐GMA and fewer persistent contacts with HEMA/TEGDMA. Hydrogen bonds formed with albumin (Figure S2D) were intermittent for all ligands; however, both warfarin and ibuprofen frequently sustained contacts, while the TEGDMA ligand never formed more than one hydrogen bond, pointing toward more dominant hydrophobic contributions.

Binding pocket retention of ligands was also analyzed through the distance of center‐of‐mass measurements (Figure 4E). Bis‐GMA and TEGDMA showed little movement from the binding cavity, with distances centered around ∼2 Å with little deviation. This shows that these two ligands do not drift too far from their original position. HEMA and trans‐chalcone experienced greater excursion distances in certain time frames up to ∼5 Å suggesting these ligands are sometimes found out of the pocket or shifted from their original position. Caffeine had intermediate values between these two groups, spending most of the simulation in the pocket but drifting out at certain times. This again supports the tight‐binding nature of Bis‐GMA. As seen with lysozyme (Figure S1E), glucosamine maintained the lowest distance overall, centered around ∼1 Å. Bis‐GMA and diacetylchitobiose stayed between ∼1 and 2 Å, indicating good binding retention. Both HEMA and TEGDMA reach distances of ∼4–5 Å, indicating they leave the pocket at certain times during the simulation. Albumin distance measurements (Figure S2E) had smaller ranges of distances for all ligands compared to BNIP and lysozyme, with ibuprofen being the closest bound ligand centering around ∼1 Å. HEMA shows a larger average distance around ∼2.5 Å, suggesting that it binds along the outskirts of the pocket. Finally, TEGDMA shows an initial increase in distance at the start of the simulation before spending the rest of the simulation within a similar range as other ligands. SASA profiles (Figure 4F) for all three systems did not show significant increases or decreases and hovered around 190–210 Å^2^. There was a slight increase in the SASA for both TEGDMA and caffeine. This suggests there is a small increase in surface area exposed, which could be caused by small positional fluctuations of protein side chains to better accommodate flexible ligands. Bis‐GMA’s complex SASA profile was relatively flat, supporting its notion of providing protein stabilization. Lysozyme SASA profiles (Figure S1F) were within tight ranges of ∼70–80 Å^2^. These small changes indicate that there are not much solvent‐induced conformational changes regardless of the ligand. Albumin SASA distributions were larger, ranging from ∼290–310 Å^2^ (Figure S2F), which makes sense as it is a multidomain protein. Despite the larger range, there was no clear expansion or contraction of surface area, which suggests that ligand binding did not affect protein stability.

### 3.4 MM/PBSA Calculation Results

Reported MM/PBSA estimates were dependent on both ligand and protein target (Table 5). Against α‐amylase, Bis‐GMA had the least mean of all tested compounds (−17.63 ± 7.60 kcal/mol), followed by trans‐chalcone (−12.09 ± 2.67 kcal/mol), HEMA (−7.89 ± 3.08 kcal/mol), TEGDMA (−7.42 ± 2.19 kcal/mol), and caffeine (−4.91 ± 3.34 kcal/mol). Against lysozyme, reference ligands diacetylchitobiose (−13.09 ± 5.21 kcal/mol) and glucosamine (−11.56 ± 2.92 kcal/mol) exhibited more negative means than Bis‐GMA (−8.61 ± 5.53 kcal/mol), TEGDMA (−6.88 ± 4.17 kcal/mol), and HEMA (−3.54 ± 1.49 kcal/mol). Tested ligands against albumin resulted in means of −24.53 ± 5.12 kcal/mol for Bis‐GMA, −22.06 ± 5.58 kcal/mol for ibuprofen, −11.26 ± 3.98 kcal/mol for warfarin, −4.90 ± 2.14 kcal/mol for HEMA, and −4.38 ± 2.08 kcal/mol for TEGDMA. Bis‐GMA had therefore the most negative reported mean among the three resin monomers for each protein target. This numerical ranking system describes only the sampled trajectories but does not assert statistically different affinities, superiority to reference ligands, or converged binding energetics.

**TABLE 5:** MM/PBSA‐calculated binding free energies (Δ*G*_binding) of dental resin monomers and reference ligands interacting with salivary proteins, derived from single 100 ns MD trajectories and thus representing exploratory computational estimates rather than statistically validated affinities.

Complex	MM/PBSA free binding energy Δ*G*_binding, kcal/mol
α‐amylase_caffeine	−4.91 ± 3.34
α‐Amylase_trans‐chalcone	−12.09 ± 2.67
α‐Amylase_Bis‐GMA	−17.63 ± 7.60
α‐Amylase_HEMA	−7.89 ± 3.08
α‐Amylase_TEGDMA	−7.42 ± 2.19
Lysozyme_glucosamine	−11.56 ± 2.92
Lysozyme_diacetylchitobiose	−13.09 ± 5.21
Lysozyme_Bis‐GMA	−8.61 ± 5.53
Lysozyme_HEMA	−3.54 ± 1.49
Lysozyme_TEGDMA	−6.88 ± 4.17
Albumin_Ibuprofen	−22.06 ± 5.58
Albumin_Warfarin	−11.26 ± 3.98
Albumin_Bis‐GMA	−24.53 ± 5.12
Albumin_HEMA	−4.90 ± 2.14
Albumin_TEGDMA	−4.38 ± 2.08

Significant frame‐to‐frame energetic fluctuations were detected for a number of complexes. Since the calculations were performed on single trajectories without replica independence or evidence of convergence, the validity of these numerical rankings is unclear. The presence of negative mean estimates does not guarantee stable binding or experimentally realistic binding free energies.

To facilitate visual comparison of monomer‐specific binding trends across the three principal protein targets, the mean predicted MM/PBSA binding free energies of Bis‐GMA, HEMA, and TEGDMA are summarized in Figure 5.

**FIGURE 5:**
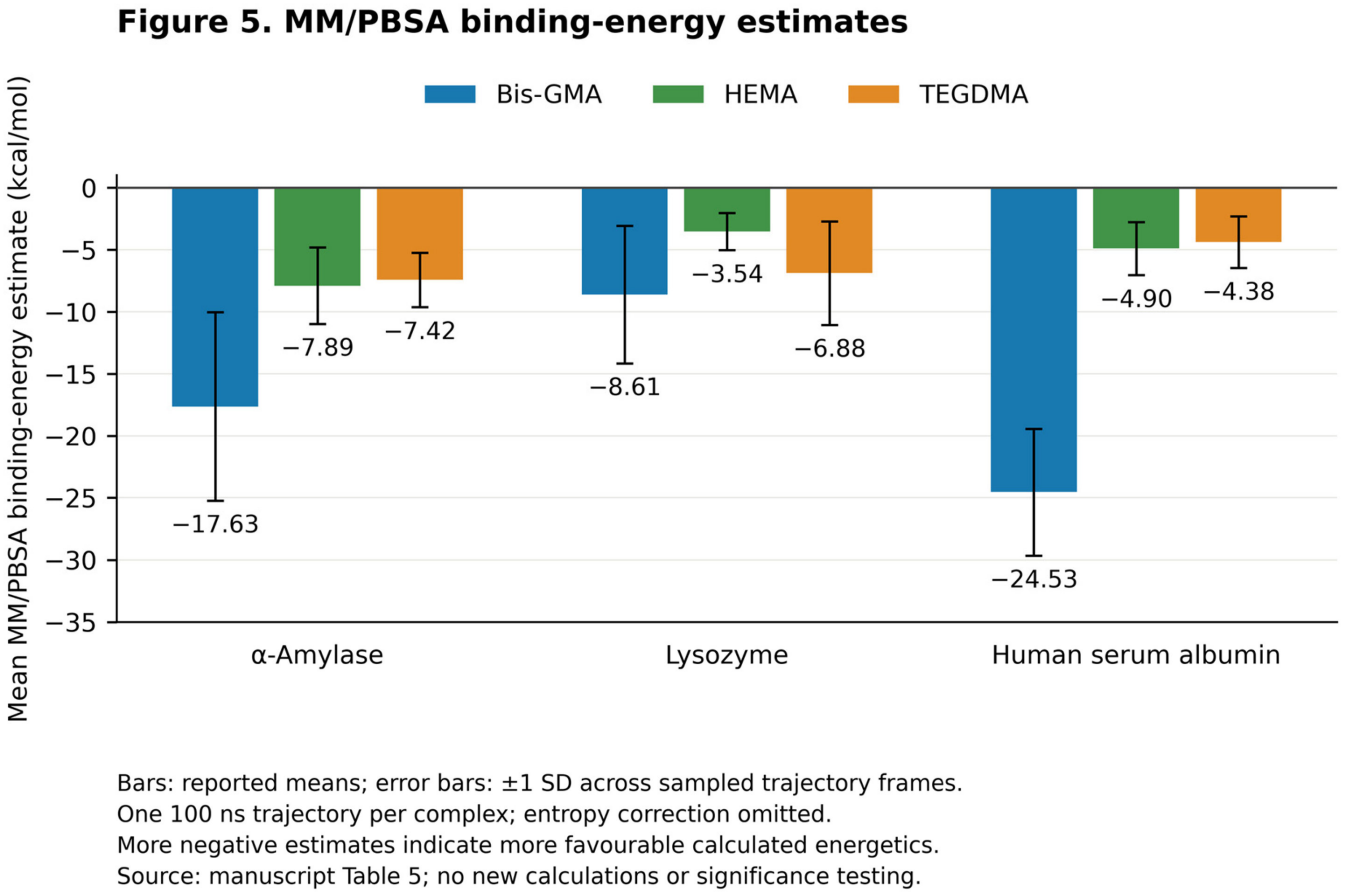
Summary of average predicted MM/PBSA binding free energies (Δ*G*binding) of Bis‐GMA, HEMA, and TEGDMA toward α‐amylase, lysozyme, and serum albumin (HSA). Bars represent the mean predicted s obtained from single 100 ns MD trajectories, and error bars indicate the standard deviation of the sampled trajectory frames used in MM/PBSA analysis. More negative values indicate more favorable predicted binding. Because independent replicate simulations were not performed, these data should be interpreted as exploratory trend‐level computational estimates rather than statistically validated differences; therefore, no significance testing or significance markers are shown.

Per‐residue decomposition heatmaps show heterogeneous energetic patterns across the α‐amylase complexes (Figure 6A–E). Negative and positive values are displayed for different protein residues, indicating favorable and unfavorable contributions, respectively, under the reported decomposition convention. Pronounced negative regions are visible around Arg195 and Asp300 in several panels, while other residues display positive contributions. These mixed patterns do not support uniformly favorable contributions from all residues associated with the docked poses. Differences in the number or intensity of colored rows were not used to rank ligand affinity, particularly because the panels have different color‐scale ranges. The heatmaps are therefore treated as exploratory descriptions of the sampled energetic environment rather than evidence of confirmed binding hotspots or stable complex formation.

**FIGURE 6:**
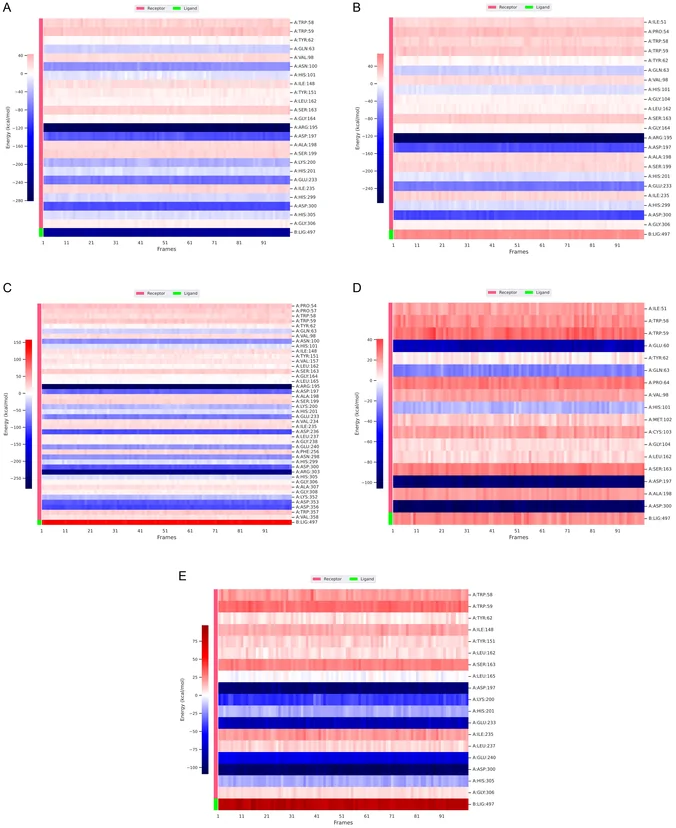
Reported per‐residue energy‐decomposition heatmaps for α‐amylase complexes with (A) caffeine, (B) trans‐chalcone, (C) Bis‐GMA, (D) HEMA, and (E) TEGDMA. Columns represent analyzed frames, and rows identify the displayed protein residues and ligand‐associated contribution. Energies are expressed in kcal/mol. Blue and red indicate negative and positive values, respectively; white represents values near zero. Color‐scale ranges differ between panels, so color intensity should not be compared directly across complexes. The pink and green side strips identify receptor and ligand rows, respectively, and do not encode energy. These exploratory maps do not establish binding affinity or simulation convergence.

The predicted binding free energies may also depend on the structure of the binding pocket. In albumin, ibuprofen and warfarin bind mainly in known hydrophobic regions. Bis‐GMA has a larger aromatic and hydrophobic surface. This allows it to form more van der Waals and hydrophobic contacts in the wide albumin pocket. Arg81, Asp107, and Asp108 also showed strong residue‐level contributions in the Bis‐GMA complex. This supports a good structural fit between Bis‐GMA and the binding site. Therefore, the favorable MM/PBSA value may be related to both ligand chemistry and the large hydrophobic nature of the albumin pocket.

Analysis of residuewise decomposition energies for lysozyme complexes (Figure S3) showed that a core cluster of energetically contributing residues around the active site region, specifically Glu_3__5_, Asp_49_, Asp_53_, Gln_58_, Ile_59_, Trp_64_, Val_99_, Gln_104_, Trp_109_, Val_110_, and Ala_111_, was conserved between complexes. Good stabilization by Glu_3__5_ and Asp_53_ for the glucosamine complex agreed with previous reports on these residues’ roles in catalysis. In contrast, diacetylchitobiose had a wider distribution of energetic residues with larger contributions from Asn_44_, Tyr_45_, Asp_49_, and Trp_64_ likely due to tighter clamping in the substrate‐binding cleft. As expected from the largest number of native‐like interactions, Bis‐GMA had the largest number of residues with favorable energetic contributions from individual residues per mutant complex, with Asp_53_, Trp_64_, Val_99_, and Trp_109_ being most frequent. Compared to Bis‐GMA, HEMA had poor per‐residue energy contributions that were largely restricted to Asp_53_ and Gln_58_. TEGDMA formed intermediate strength contacts with Glu_3__5_, Asp_49_, Trp_64_, and Gln_104_.

For lysozyme, glucosamine, and diacetylchitobiose, showed more favorable mean binding free energies than the resin monomers. This may be related to the carbohydrate‐binding nature of the lysozyme cleft. Diacetylchitobiose also interacted with a wider range of residues. Bis‐GMA showed an intermediate energy profile. HEMA and TEGDMA showed less favorable values. These reference ligands help in comparing the relative binding of the monomers. However, the results do not prove direct competitive inhibition.

The ligand‐associated row is predominantly negative for caffeine (Figure 6A) and positive for trans‐chalcone, Bis‐GMA, HEMA, and TEGDMA (Figure 6B–E). This contrasts with the negative mean total MM/PBSA estimates reported in Table 5 and requires cautious interpretation. A ligand‐associated decomposition row is not equivalent to the total complex binding‐energy estimate; nevertheless, its positive values should not be disregarded when discussing the calculated energetic balance. Complete numerical decomposition would be required to reconcile these contributions quantitatively with the reported totals. Accordingly, Figure 6 is not used as independent confirmation of the affinity ranking in Table 5. Contacts identified by docking also do not necessarily correspond to favorable energetic contributions throughout MD sampling.

Energy analysis was also conducted for the albumin complexes (Figure S4). Unlike α‐amyase and lysozyme, interacting hotspots were not found localized to one region but instead spanned a broad hydrophobic pocket with frequent recurrence of Leu_31_, Gln_32_, Ala_78_, Arg_81_, Thr_83_, Tyr_84_, Gly_85_, Phe_87_, Gln_104_, His_105_, Lys_106_, Asp_107_, Asp_108_, Asn_109_, and Pro110, as well as more distal residues Lys466 and Thr467. Binding of ibuprofen was found to be most stabilized by Arg_81_, Tyr_84_, and Asp_107_, which reside within known drug‐binding domains of albumin23. Similar interacting hotspots were found for warfarin, with increased interaction of Gln_32_ and His_105_. The Bis‐GMA complex exhibited particularly strong energetic minima at Arg_81_, Asp_107_, and Asp_108_, which supports its notably exergonic binding free energy and suggests electrostatic anchoring within the pocket. HEMA and TEGDMA were observed to produce weaker, more dispersed energetic landscapes upon simulation. These broad interactions were stabilized almost exclusively by Gln104, Lys106, and Asp108, which do not reside within the hydrophobic pocket, suggesting only fleeting interactions. Taken together, per residue energy landscapes reveal that Arg195 and Asp300 serve dominant roles in stabilization within α‐amylase, Glu_35_ and Asp_53_ within lysozyme, and Arg_81_ and Asp_107/108_ within albumin. In all proteins studied, Bis‐GMA formed the largest contact surfaces with the most negative residuewise energetic contributions, which supports its potent stabilizing effects. Contrarily, HEMA formed few energetic hotspots, which correlate with its weak and transient binding.

## 4 Discussion

In this study, we carried out a detailed molecular‐level investigation of interactions between commonly used dental resin monomers and the three major salivary proteins using molecular docking, DFT, MD simulations, MM/PBSA free energy calculations, and per‐residue energy decomposition. Overall, our results highlight that the physicochemical properties of monomers (such as size, polarity, and conformational flexibility) play determining roles in their recognition and binding with these proteins. These findings are of biological significance because residual unpolymerized resin monomers are released from restorations and come into direct contact with saliva and oral tissues immediately after placement. Due to incomplete polymerization, dental methacrylate‐based restorative materials have been reported to release residual monomers such as Bis‐GMA, HEMA, and TEGDMA into the oral environment [62, 63]. These compounds can diffuse through saliva, dentin, and soft tissues, potentially eliciting local or systemic biological responses. Previous studies have shown that resin monomers may induce oxidative stress, inflammatory signaling, and cytotoxic effects depending on their concentration and chemical structure [64, 65]. Therefore, understanding how these monomers interact with salivary proteins is critical as protein binding may influence their bioavailability, transport, and biological persistence.

This suggests a clinically relevant sequence. Residual monomers may be released from resin restorations into the saliva. They can then come into contact with salivary proteins. Some monomers may bind to these proteins. This may affect their local availability and biological behavior.

These interactions may indirectly contribute to some reported effects of methacrylate monomers. These include oxidative stress, inflammation, mucosal irritation, and hypersensitivity. However, this computational study does not prove that protein binding causes these effects. Further experimental and clinical studies are needed [66].

The toxicological effects of the three monomers should also be considered when interpreting their protein interactions. Bis‐GMA is a relatively hydrophobic and high‐molecular‐weight monomer. Its unpolymerized form has been linked with cytotoxic and inflammatory effects.

HEMA is smaller and more hydrophilic. It can easily diffuse through aqueous environments and dentin. In this study, HEMA showed weaker predicted protein binding. However, its greater mobility may increase cellular exposure. This may contribute to its cytotoxic effects.

TEGDMA has also been linked with concentration‐dependent cytotoxicity. It may cause oxidative stress and disturb the cellular redox balance. It may also affect immune responses.

Therefore, stronger predicted protein binding does not necessarily mean greater toxicity. Toxicity also depends on the monomer concentration, release, diffusion, cellular uptake, metabolism, and exposure time. The present computational study only evaluates molecular interactions with proteins. This does not provide an independent toxicological assessment.

No new monomer candidates were developed in this study. Therefore, the toxicity assessment was limited to the three clinically relevant monomers included in the analysis.

The data gained from DFT analysis can help to rationalize disparate binding trends exhibited in the docking and MD simulation results. Bis‐GMA displayed the largest HOMO–LUMO gap, suggesting relatively low electronic chemical reactivity; however, its large aromatic structure and bulky hydrophobic surface area enabled maximization of van der Waals, π–alkyl, and π–sigma interactions within hydrophobic pockets of proteins. This is consistent with Bis‐GMA producing the strongest binding profiles for both docking and MM/PBSA analyses despite possessing a lower dipole moment relative to TEGDMA. HEMA had a lower HOMO–LUMO gap than Bis‐GMA and possessed polar functional groups that would facilitate localized polar contacts and hydrogen‐bond‐capable interactions. However, due to its small structure and lack of hydrophobic surface area, the monomer had fewer stabilizing contacts, which could account for weaker docking scores and higher variation in RMSD trajectories. TEGDMA displayed the lowest HOMO–LUMO gap with the highest dipole moment, implying higher electronic softness and polarity. These characteristics may allow for polar interactions with protein residues, but the flexible linear structure may afford conformational mobility.

Overall, the DFT, MD, and MM/PBSA results showed a similar trend.Bis‐GMA had the largest HOMO–LUMO gap and relatively low polarity. This suggests greater electronic rigidity. Its large aromatic structure also allowed several hydrophobic and van der Waals interactions within the protein‐binding pockets. During MD simulations, Bis‐GMA showed relatively small conformational changes. It also showed more favorable predicted MM/PBSA binding energies.

HEMA behaved differently. It formed fewer stabilizing interactions and showed greater changes during the MD simulations. Its predicted binding free energies were also less favorable.

TEGDMA had the smallest HOMO–LUMO gap and the highest dipole moment. This suggests greater electronic softness and polarity. Its flexible linear structure may also allow it to adjust more easily within the binding pocket. This was reflected in its intermediate‐binding behavior.

Overall, these findings suggest that electronic properties, molecular flexibility, and predicted binding stability are closely related. They should therefore be considered together when interpreting monomer–protein interactions.

In general, Bis‐GMA displayed the best binding affinities among all tested monomers in all computational studies. In the docking study, Bis‐GMA achieved numerous atomic contacts, mainly involving hydrophobic contacts and carbon–oxygen bonds. MD simulations showed that Bis‐GMA had the best conformational stability, indicated by low RMSD values, small structural compactness, and high frequency of residues remaining within the protein cavity. This was further illustrated by MM/PBSA results where Bis‐GMA outperformed ibuprofen in terms of binding free energy for albumin. This trend was expected due to Bis‐GMA’s bulky aromatic structure, allowing for van der Waals interactions and hydrophobic fitting in protein pockets. Enhanced aromatic interactions have been linked with increased protein affinities in previous studies of ligand–protein recognition [67]. Albumin emerged as a particularly favorable binding partner for Bis‐GMA, suggesting a potential carrier‐like role in the oral cavity. However, given albumin’s known promiscuity, future studies employing blind docking or ensemble approaches would be valuable to confirm these specific binding modes and explore potential alternative interaction sites [68, 69]. Strong albumin binding can prolong molecular half‐life and modulate tissue distribution, raising the possibility that hydrophobic dental monomers could persist longer in saliva than previously assumed. This mechanism may partly explain why certain resin‐derived compounds have been detected in biological fluids after restorative procedures [70].

On the other hand, HEMA had lower binding energies, fewer contributions from stabilizing residues, and exhibited more structural fluctuations during MD simulations. This finding correlates well with its lower molecular weight and higher polarity, leading to less hydrophobic complementarity but allowing transient hydrogen bonding interactions. Experimentally, HEMA’s diffusion capabilities have been reported and have been related to greater pulpal penetration and cytotoxicity even with weaker protein binding [71]. Therefore, lower affinity interactions do not necessarily indicate lower biological potential for harm but may allow more rapid diffusion through biological membranes. This is supported by our DFT studies. While HEMA does contain polar groups and a smaller HOMO–LUMO gap than Bis‐GMA, its low‐molecular weight does not allow contact with as many protein residues at one time. This could indicate that while HEMA may make contact through local polar interactions, it may not fill and stabilize large protein pockets like Bis‐GMA.

TEGDMA fell in between Bis‐GMA and HEMA in terms of having intermediate polarity and significant conformational freedom. Flexible ligands can often adapt to multiple binding topologies, which can explain why TEGDMA had spread out residue‐level energy contributions. However, conformational freedom can also lead to increased nonspecific interactions with proteins, which has been commonly reported with methacrylate monomers [64, 72]. Studies have shown TEGDMA affects cellular redox potential and immune mechanisms beyond cytotoxicity alone [64]. Experimental work has further shown that TEGDMA can alter the cellular redox balance and interfere with immune responses, emphasizing the need to understand its molecular interactions beyond simple toxicity assays [65]. Taken together, our MD‐derived structural descriptors suggest binding by monomers does not significantly unfold salivary proteins on a global scale (RoG and SASA values do not drastically change); rather, small localized adaptations likely occur in loop regions to accommodate ligand binding. Protein–ligand recognition is often described as a dynamic process in which conformational selection and induced fit permit proteins the structural resilience to interact with chemically heterogeneous ligands without significantly altering global stability [73]. TEGDMA’s large dipole moment and low HOMO–LUMO gap energy indicate that it has higher polarity and less electronic rigidity, potentially allowing for more polar interactions with protein residues. Its linear and flexible nature may also mean there is less continuous pocket occupancy when bound, which may attribute to why it behaved intermediate between Bis‐GMA and HEMA.

It is important to keep the limitations of single‐trajectory sampling in mind when considering the MD and MM/PBSA results. While the 100 ns trajectories offer a qualitative comparison of ligand retention, flexibility, and predicted energetics, they are by no means statistically reproducible. Variability in RMSD, hydrogen‐bond lifetime, ligand‐distance trajectories, or MM/PBSA free energies may arise from initial condition biases and sampling that is unique to each trajectory. As such, these trends should be viewed as hypothesis‐generating and confirmed through replicate simulations and experiment.

## 5 Limitations and Clinical Considerations

Several limitations of this study should be considered. First, this study was performed using computational analysis such as molecular docking studies, MD simulations, MM/PBSA calculations, and per‐residue energy decomposition analysis. Despite their frequent use to predict protein‐ligand interactions, in silico studies cannot completely mimic physiological conditions found in the oral cavity, such as salivary flow, enzymatic degradation, temperature changes, pH changes, and competition from other salivary proteins and biomolecules, which may impact the stability of these interactions. Furthermore, force‐field limitations, time scale of simulations, and implicit solvent model may impact the calculated absolute binding energies. Experimental validation using methods like isothermal titration calorimetry, surface plasmon resonance, or protein‐binding studies in vitro is required to confirm the findings of this study.

Each complex was represented by a single 100 ns production trajectory. Longer simulations and independent replicate trajectories were not performed, and convergence of the binding‐energy estimates has not been established. The residue‐decomposition heatmaps do not resolve this sampling limitation or demonstrate stable ligand retention. Consequently, the reported MM/PBSA means and residue‐level patterns remain exploratory, and the ligand rankings may change with additional sampling.

This limitation can be overcome by performing experimental validation in a stepwise manner. Initial validation should involve direct measurement of monomer–protein binding using biophysical techniques (e.g., surface plasmon resonance, isothermal titration calorimetry, fluorescence quenching, microscale thermophoresis) to assess predicted binding affinity, binding kinetics, and thermodynamic parameters. Structural/conformational impacts to salivary proteins could be subsequently measured using spectroscopic techniques (circular dichroism spectroscopy, intrinsic fluorescence spectroscopy, and Fourier transform infrared spectroscopy). Functional assays can then be conducted to assess whether monomer binding inhibits protein function (e.g., α‐amylase enzymatic assays, lysozyme antibacterial/lytic assays, and albumin ligand‐displacement assays). This approach can then be expanded to artificial saliva and human saliva samples to account for competitive binding interactions, salivary flow, pH fluctuations, and the presence of additional salivary proteins. Performing validation in a stepwise manner would allow for computational predictions to be tested under biologically relevant conditions and could enhance clinical translation.

Clinically speaking, based on predicted binding affinities, some monomers, including Bis‐GMA, may bind to salivary proteins with high affinity. However, effects on protein conformation, enzyme activity, acquired pellicle formation, or biocompatibility cannot be ruled out until proven otherwise through experimentation. Clinically extrapolating this data must be done carefully, though, as leached concentrations are usually low and may not even persist in the oral environment for an extended period. Other factors unique to each patient, such as saliva composition, oral hygiene, age of restoration, and polymerization/shrinkage, can also affect in vivo outcomes. Additionally, the synergistic or antagonistic effects between multiple released compounds were not assessed.

Another limitation is that each protein–monomer complex was only subjected to one 100 ns MD simulation. While this length was adequate for generating valuable comparative information regarding relative structure stability, ligand retention, and interaction persistence, replicate MD simulations were not performed. As such, the MD trends observed herein should be considered supportive/hypothesis‐generating rather than representative of true converged dynamic behavior. Replicate simulations, longer trajectories, and experimental validation are warranted to verify predicted interaction stability.

In the α‐amylase system, HEMA, trans‐chalcone, and caffeine showed larger changes in the RMSD and ligand distance. This suggests that 100 ns may not be enough to fully assess their conformational behavior. Future studies should use longer simulation times. Independent replicate simulations would also be useful. These studies could show whether the complexes eventually reach stable and persistent conformations.

Another limitation worth mentioning is that of the chosen protein structures; statherin was chosen using an AlphaFold predicted structure rather than an experimentally solved PDB structure. AlphaFold structures can be helpful when no structures are available; however, predicted structures may not account for bound ligands, local pocket flexibility, or solvent‐induced binding‐site plasticity. Docking results with statherin should be viewed with an added grain of salt for this reason.

Another limitation concerns the selection of docking regions. For the enzymatic proteins, docking was performed only in catalytic or experimentally/functionally annotated active‐site regions. However, a number of salivary proteins assessed in this study are nonenzymatic or promiscuous proteins that may be capable of binding small molecules via multiple surface‐accessible pockets or via nonspecific interaction sites. For example, albumin may bind a variety of ligands via several hydrophobic cavities as well as electrostatic anchoring regions. Thus, limiting docking to selected binding regions may have precluded identification of alternate monomer‐binding modes. Blind, ensemble, and comparative docking across multiple predicted pockets should be performed in future assessments for carrier and pellicle‐associated proteins.

Another limitation is the absence of complete natural substrates in the simulation set. For α‐amylase, compounds such as maltoheptaose could provide a useful physiological comparison. For lysozyme, N‐acetylglucosamine oligomers such as NAG_3_ could serve a similar role. The reference ligands used in this study were chosen as protein‐specific controls. They were not intended to represent direct competition with natural substrates. Future studies should include these natural substrates. They should be tested using the same docking, replicate MD, and MM/PBSA methods. This would allow a more direct assessment of possible competitive interactions.

Another important limitation is that replicate MD simulations were not performed. Only one 100 ns trajectory was run for each protein–ligand complex, and MM/PBSA calculations were based on these single trajectories. As such, the MD and MM/PBSA results presented herein are best thought of as preliminary computational approximations and should not be viewed as statistically reproducible estimates of binding stability or binding free energy. In the future, we plan to perform replicate simulations starting from the same crystal structure but with different initial velocity seeds, and we will run longer trajectories with formal statistical comparisons made across independent runs to ensure convergence and reproducibility.

Longer simulations and independent replicate runs are needed to confirm whether the observed energy changes eventually become stable. Analysis of only the final part of a simulation, such as the last 10 ns, would be useful only if the system had already reached a stable and well‐equilibrated state. For this reason, the present study considers the full simulation trajectory rather than selecting a visually stable final segment to support stronger binding. Future studies could also examine more protein targets and alternative binding sites. The most promising complexes could then be tested using longer and repeated MD simulations.

## 6 Conclusion and Future Works

The findings of this study are based entirely on computational analyses. These included molecular docking, DFT calculations, MD simulations, MM/PBSA analysis, and per‐residue energy decomposition. The results have not yet been confirmed by laboratory experiments, enzyme assays, or cytotoxicity studies. Among the tested monomers, Bis‐GMA showed the most favorable overall interaction with the selected salivary proteins. HEMA showed weaker and more variable interactions. TEGDMA generally showed intermediate behavior. Albumin, α‐amylase, and lysozyme appeared to be important potential targets for these monomers. However, the differences observed in this study should be considered as computational trends. They should not be taken as proof of statistically different binding affinities.

The biological effects of these predicted interactions are still uncertain. Monomer binding may affect how these compounds are retained or distributed in the oral environment. It may also influence protein function or local biocompatibility. However, these effects cannot be confirmed by computational simulations alone. Experimental studies are needed to verify the findings. Methods such as SPR, ITC, enzyme‐activity assays, protein‐binding studies, and cell‐based tests could be used. These experiments would help determine whether the predicted interactions occur under physiological conditions. They would also clarify whether the interactions have any functional or toxicological importance.

## Author Contributions

**Ravinder Saini:** conceptualization, methodology, supervision, project administration, and funding acquisition. **Fahad Hussain Alhamoudi:** conceptualization, methodology. **Rayan Ibrahim H. Binduhayyim:** data curation, formal analysis. **Abdulkhaliq Ali F. Alshadidi:** data curation, formal analysis. **Abdullah Hassan A. Alshehri:** investigation, resources. **Abdulmajeed Okshah:** data curation, formal analysis. **Ahid Amer Alshahrani:** writing, reviewing, and editing. **Mohamed Saheer Kuruniyan:** investigation and resources. **Vishwanath Gurumurthy:** investigation and resources. **Masroor Ahmed Kanji:** writing, reviewing, and editing. **Artak Heboyan:** validation, formal analysis, writing, reviewing, and editing.

## Funding

The authors extend their appreciation to the Deanship of Research and Graduate Studies at King Khalid University for funding this work through the Large Research Project under grant number RGP2/405/47.

## Conflicts of Interest

The authors declare no conflicts of interest.

## Supporting information

Molecular Docking Results

Molecular Interactions

Supplementary Material

## Data Availability

The computational input files, available topology files, DFT workflow scripts, and analysis materials supporting this study are available from the corresponding author upon reasonable request. A public repository was not established for the present study. Future work will prioritize deposition of computational input files and analysis scripts in an open repository to facilitate reproducibility.
