# Supplementary material for: Molecular Recognition of Dental Resin Monomers by Salivary Proteins: Integrated Docking, DFT, Molecular Dynamics, and MM/PBSA Analysis
Source: ChemistryOpen. 2026 Nov 2;15(11):e70332. doi: 10.1002/open.70332 (PMC13728541; doi:10.1002/open.70332)

**Supplementary Data 1 - Molecular Docking Results**
**Supplementary Data 2 - Molecular Interactions**

# Molecular Recognition of Dental Resin Monomers by Salivary Proteins: Integrated Docking, DFT, Molecular Dynamics, and MM/PBSA Analysis

Ravinder S Saini[1], Fahad Hussain Alhamoudi[2], Rayan Ibrahim H Binduhayyim[3], Abdulkhaliq Ali F Alshadidi[4], Abdullah Hassan A. Alshehri[5], Abdulmajeed Okshah[6], Ahid Amer Alshahrani[7], Mohamed Saheer Kuruniyan[8], Vishwanath Gurumurthy[9], Masroor Ahmed Kanji[10], Artak Heboyan[11, 12, 13]

**Supplementary Figure 1. Molecular dynamics profiles of lysozyme complexes over 100 ns.** Comparative analysis of lysozyme bound to the dental resin monomers Bis-GMA, HEMA, and TEGDMA and the reference ligands glucosamine and diacetylchitobiose. Panels show (A) root-mean-square deviation (RMSD), (B) residue-wise root-mean-square fluctuation (RMSF), (C) radius of gyration (RoG), (D) number of protein–ligand hydrogen bonds, (E) ligand–binding-site center-of-mass distance, and (F) solvent-accessible surface area (SASA). Individual complexes are identified in the legends.

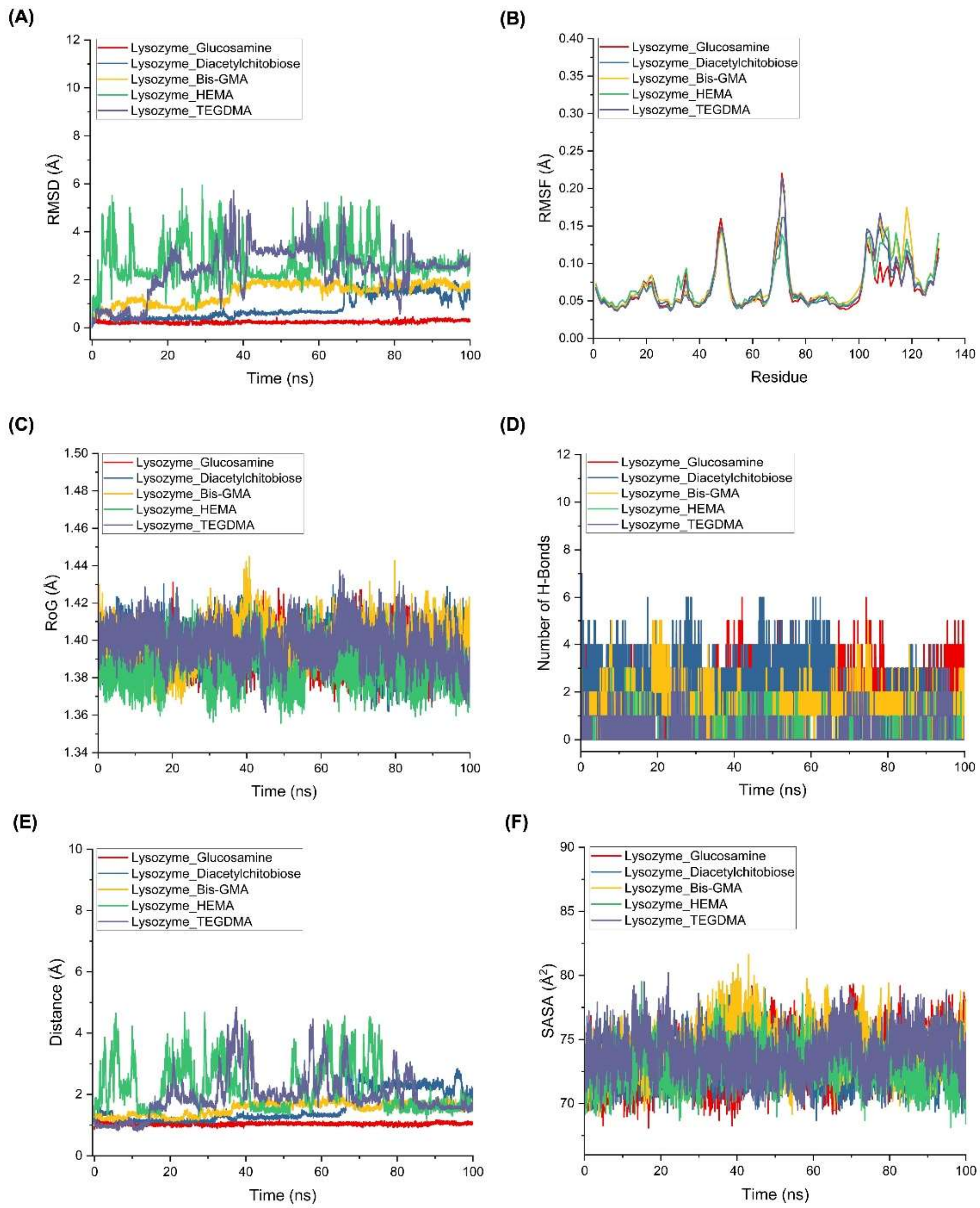

**Supplementary Figure 2. Molecular dynamics profiles of albumin complexes over 100 ns.** Comparative analysis of albumin bound to the dental resin monomers Bis-GMA, HEMA, and TEGDMA and the reference ligands ibuprofen and warfarin. Panels show (A) root-mean-square deviation (RMSD), (B) residue-wise root-mean-square fluctuation (RMSF), (C) radius of gyration (RoG), (D) number of protein–ligand hydrogen bonds, (E) ligand–binding-site center-of-mass distance, and (F) solvent-accessible surface area (SASA). Individual complexes are identified in the legends.

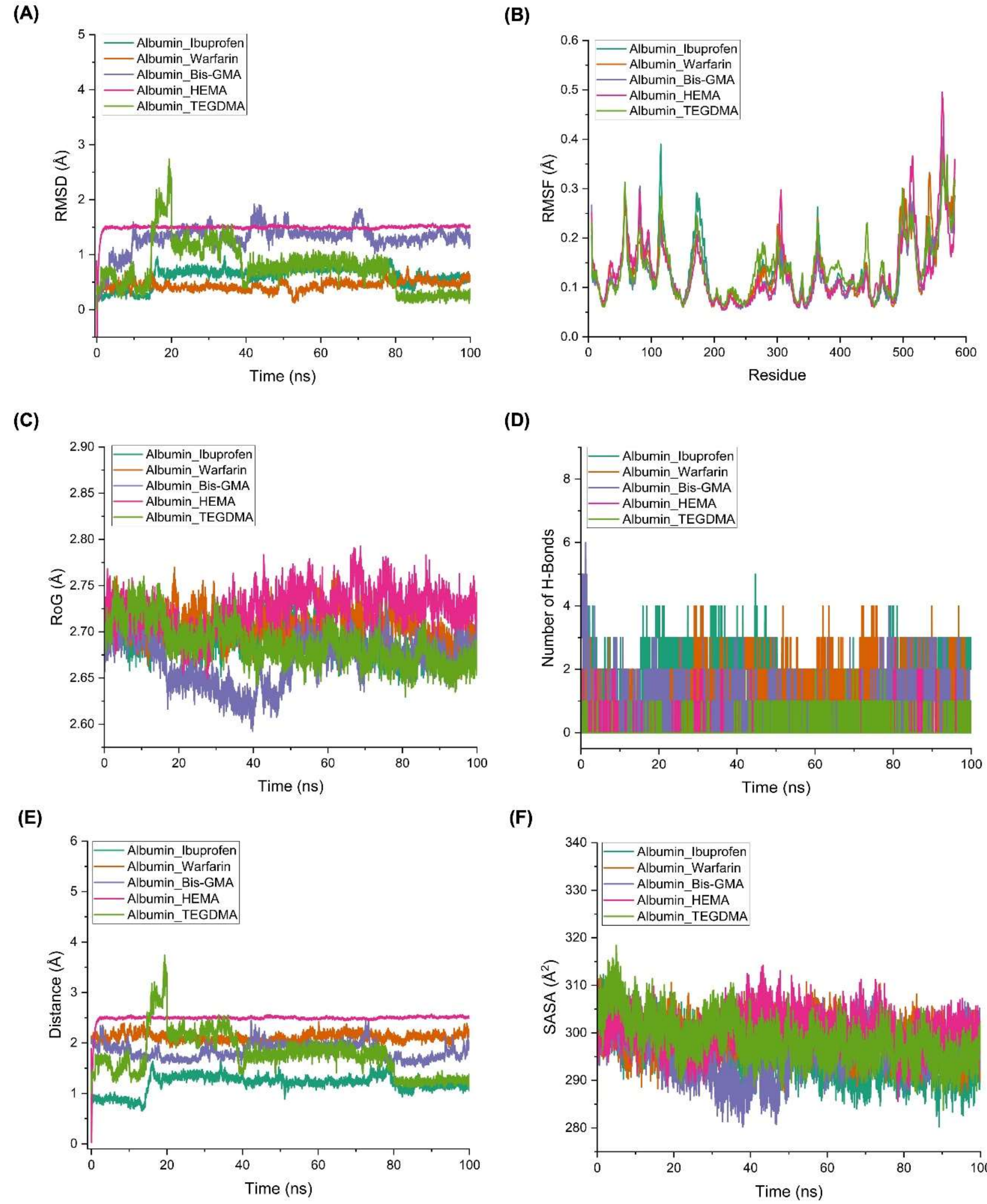


**Supplementary Figure 3. Residue-wise energy decomposition heatmaps for lysozyme–ligand complexes.** Heatmaps depict energy contributions across sampled molecular dynamics frames for lysozyme complexed with (A) glucosamine, (B) diacetylchitobiose, (C) Bis-GMA, (D) HEMA, and (E) TEGDMA. Columns represent sampled frames, and rows identify protein residues and the ligand.

Energies are expressed in kcal/mol; blue indicates negative contributions and red indicates positive contributions, according to the color scale accompanying each panel. Side annotations distinguish receptor residues from the ligand.

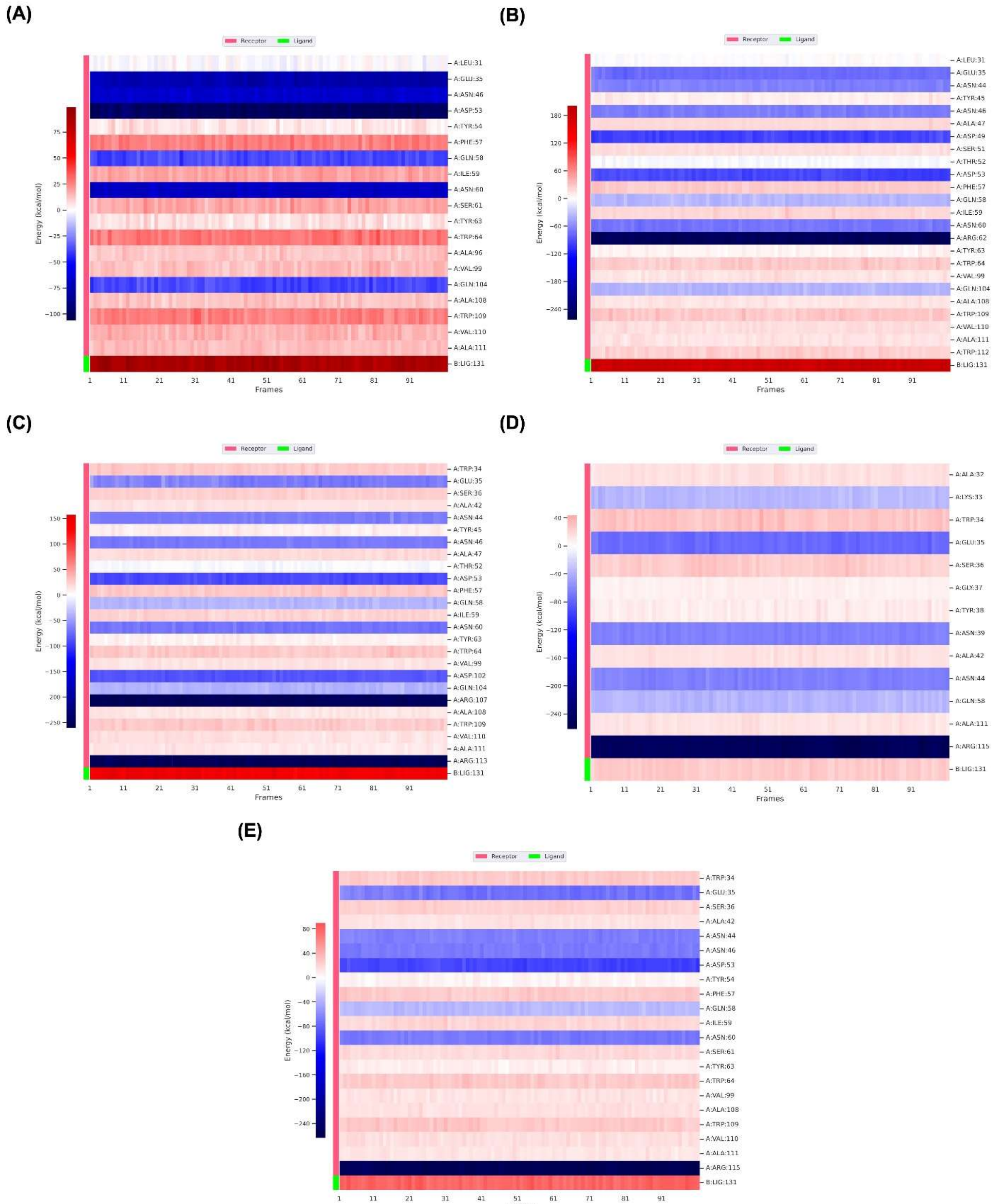


**Supplementary Figure 4. Residue-wise energy decomposition heatmaps for albumin–ligand complexes.** Heatmaps depict energy contributions across sampled molecular dynamics frames for albumin complexed with (A, B) the reference ligands ibuprofen and warfarin, (C) Bis-GMA, (D) HEMA,

and (E) TEGDMA. Columns represent sampled frames, and rows identify protein residues and the ligand. Energies are expressed in kcal/mol; blue indicates negative contributions and red indicates positive contributions, according to the color scale accompanying each panel. Side annotations distinguish receptor residues from the ligand.

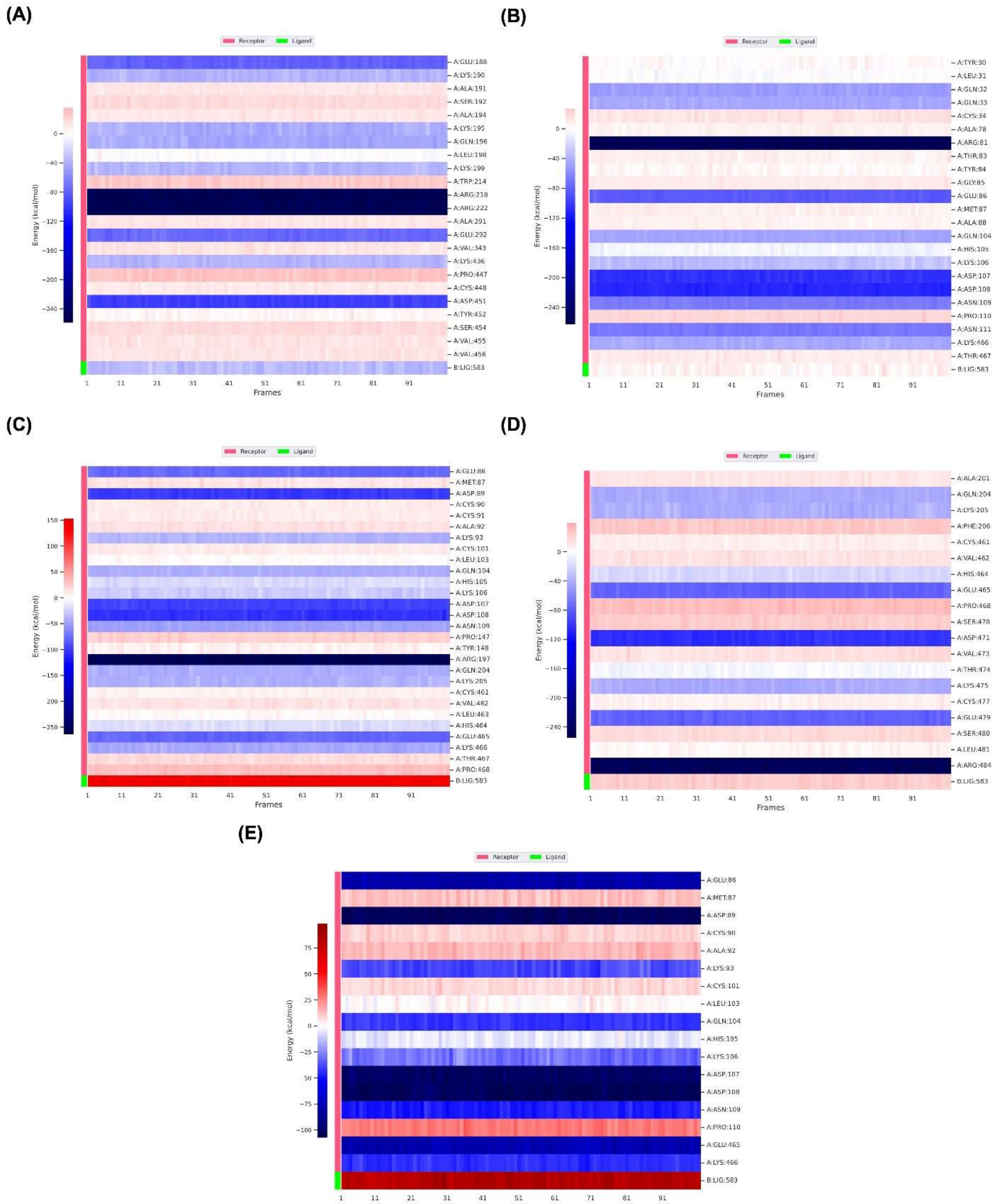

Supplement: Supplementary file 1 [file OPEN-15-e70332-s002.pdf]
